# Rapid Virucidal Activity of Japanese *Saxifraga* Species-Derived Condensed Tannins against SARS-CoV-2, Influenza A Virus, and Human Norovirus Surrogate Viruses

**DOI:** 10.1128/aem.00237-23

**Published:** 2023-05-15

**Authors:** Toshihiro Murata, Dulamjav Jamsransuren, Sachiko Matsuda, Haruko Ogawa, Yohei Takeda

**Affiliations:** a Division of Pharmacognosy, Tohoku Medical and Pharmaceutical University, Sendai, Miyagi, Japan; b Department of Veterinary Medicine, Obihiro University of Agriculture and Veterinary Medicine, Obihiro, Hokkaido, Japan; c Research Center for Global Agromedicine, Obihiro University of Agriculture and Veterinary Medicine, Obihiro, Hokkaido, Japan; University of Queensland

**Keywords:** condensed tannin, influenza A virus, norovirus, SARS-CoV-2, *Saxifraga*, virucidal activity

## Abstract

Severe acute respiratory syndrome coronavirus 2 (SARS-CoV-2), influenza A virus (IAV), and norovirus are global threats to human health. The application of effective virucidal agents, which contribute to the inactivation of viruses on hands and environmental surfaces, is important to facilitate robust virus infection control measures. Naturally derived virucidal disinfectants have attracted attention owing to their safety and eco-friendly properties. In this study, we showed that multiple Japanese *Saxifraga* species-derived fractions demonstrated rapid, potent virucidal activity against the SARS-CoV-2 ancestral strain and multiple variant strains, IAV, and two human norovirus surrogates: feline calicivirus (FCV) and murine norovirus (MNV). Condensed tannins were identified as active chemical constituents that play a central role in the virucidal activities of these fractions. At a concentration of 25 μg/mL, the purified condensed tannin fraction *Sst*-2R induced significant reductions in the viral titers of the SARS-CoV-2 ancestral strain, IAV, and FCV (reductions of ≥3.13, ≥3.00, and 2.50 log_10_ 50% tissue culture infective doses [TCID_50_]/mL, respectively) within 10 s of reaction time. Furthermore, at a concentration of 100 μg/mL, *Sst*-2R induced a reduction of 1.75 log_10_ TCID_50_/mL in the viral titers of MNV within 1 min. Western blotting and transmission electron microscopy analyses revealed that *Sst*-2R produced structural abnormalities in viral structural proteins and envelopes, resulting in the destruction of viral particles. Furthermore, *Saxifraga* species-derived fraction-containing cream showed virucidal activity against multiple viruses within 10 min. Our findings indicate that *Saxifraga* species-derived fractions containing condensed tannins can be used as disinfectants against multiple viruses on hands and environmental surfaces.

**IMPORTANCE** SARS-CoV-2, IAV, and norovirus are highly contagious pathogens. The use of naturally derived components as novel virucidal/antiviral agents is currently attracting attention. We showed that fractions from extracts of *Saxifraga* species, in the form of a solution as well as a cream, exerted potent, rapid virucidal activities against SARS-CoV-2, IAV, and surrogates of human norovirus. Condensed tannins were found to play a central role in this activity. The *in vitro* cytotoxicity of the purified condensed tannin fraction at a concentration that exhibited some extent of virucidal activity was lower than that of 70% ethanol or 2,000 ppm sodium hypochlorite solution, which are popular virucidal disinfectants. Our study suggests that *Saxifraga* species-derived fractions containing condensed tannins can be used on hands and environmental surfaces as safe virucidal agents against multiple viruses.

## INTRODUCTION

Severe acute respiratory syndrome coronavirus 2 (SARS-CoV-2), influenza A virus (IAV), and norovirus are important pathogenic viruses that cause large numbers of infections and deaths worldwide. SARS-CoV-2 is an enveloped, positive-sense, single-stranded RNA virus belonging to the genus *Betacoronavirus* in the family *Coronaviridae*. SARS-CoV-2 causes various clinical symptoms, especially respiratory ones. It is transmitted mainly by the inhalation of droplets, although direct contact also appears to be a route of transmission (1). Although several therapeutic drugs and vaccines have been developed, the spread of SARS-CoV-2 infection is not fully controlled, and the emergence of variant strains with altered antigenicity and transmissibility makes ending the pandemic difficult (1–4). IAV is an enveloped, negative-sense, single-stranded RNA virus that belongs to the genus *Alphainfluenzavirus* in the family *Orthomyxoviridae*. IAV causes fever and respiratory symptoms, and a large number of people are affected by global seasonal epidemics of this virus. Because of the occurrence of genetic reassortment, new strains with significantly altered antigenicity have emerged and have caused influenza pandemics repeatedly over the last 100 years (5). Although therapeutic drugs and vaccines for influenza are available, the emergence of drug-resistant strains and the risk of low vaccination efficacy, which arises from mismatches between the antigenicity of the field strain and that of the vaccine strain, remain challenges in the control of IAV (5, 6). Norwalk virus, a human norovirus (HuNoV), is a nonenveloped, positive-sense, single-stranded RNA virus that belongs to the genus *Norovirus* in the family *Caliciviridae*. This virus is an important pathogen that causes acute gastroenteritis in people of all age groups. It is estimated that about one-fifth of acute enteritis cases worldwide are caused by this virus each year (7). Because vaccines and therapeutic drugs for HuNoV have not yet been developed, other infection prevention measures are needed to control this highly infectious agent. One such control measure is the inactivation of viruses on hands and environmental surfaces. In SARS-CoV-2 and IAV infections, although direct (and possibly indirect) contact transmission is considered a minor mode of transmission (1, 5), hand hygiene is still recognized as an important measure (1, 5; https://www.cdc.gov/flu/professionals/infectioncontrol/healthcaresettings.htm). HuNoV-infected patients shed a large number of viral particles in their stools and vomitus, and these viruses are stable in the environment. Because it appears that as few as 10 infectious viral particles may be able to cause infection, both direct and indirect transmissions occur easily, resulting in frequent outbreaks. Therefore, effective inactivation of infectious viruses is crucial to prevent the spread of HuNoV infection (8).

The use of naturally derived antiviral and virucidal components is currently attracting attention as the basis for novel antiviral drugs and safe, eco-friendly virucidal agents. Some plant-derived extracts, essential oils, isolated terpenoids, lignans, alkaloids, and phenols, such as flavonoids, tannins, and coumarins, have been reported to show virucidal and antiviral activities. These activities include blocking virus absorption, inhibiting virus entry, and suppressing virus proliferation in infected cells (9, 10). In a previous study, we reported that a pyrogallol-enriched fraction obtained from the extract of the medicinal plant Saxifraga spinulosa showed potent virucidal activity against SARS-CoV-2 (A lineage; ancestral strain), IAV, and two HuNoV surrogate viruses: feline calicivirus (FCV) and murine norovirus (MNV) (11). The genus *Saxifraga* contains over 400 morphologically and cytologically diverse species (12). Saxifraga stolonifera Curtis, S. fortunei Hook., S. nipponica Makino, S. cortusifolia Siebold & Zucc. (a synonym of S. serotina Sipliv.), and S. rebunshirensis Sipliv. are distributed across East Asia, including Japan, and are cultivated as ornamental plants. *S*. *stolonifera* and *S*. *fortunei* are edible and medicinal plants that have been used as diuretic and antibacterial drugs and for treating inflammatory symptoms such as eczema, swelling, and hemorrhoids in Japanese and Chinese medicine (13). The chemical constituents of *S*. *stolonifera* include flavonoids, bergenin, arbutin, and condensed tannins (proanthocyanidins) (13–15). However, the aforementioned previous study demonstrated the virucidal activity of only a single *Saxifraga* species, *S. spinulosa*; therefore, whether such virucidal activity is common to other *Saxifraga* species remains elusive. In addition, it is unknown which compounds in *Saxifraga* species exhibit virucidal activities. Therefore, in this study, we evaluated the virucidal activities of fractions derived from five Japanese *Saxifraga* species against SARS-CoV-2 (ancestral and multiple variant strains), IAV, FCV, and MNV. We also attempted to identify the compounds responsible for the strong virucidal activity of these fractions.

## RESULTS

### *Saxifraga*-derived samples.

**FIG 1:**
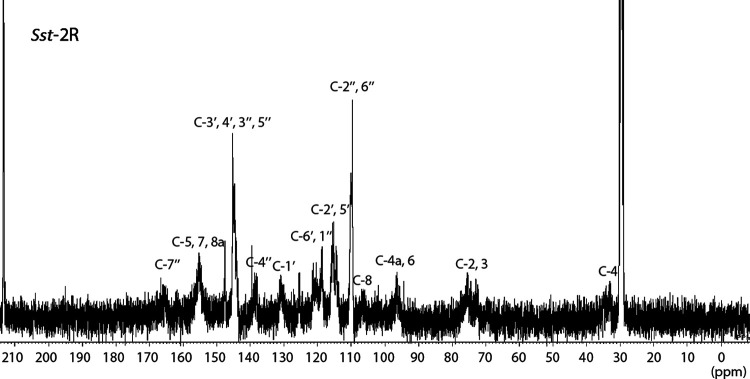
^13^C NMR spectrum of *Sst*-2R [acetone-*d*_6_-D_2_O (1:1), 100 MHz].

Fractionation and chemical investigations of extracts from five Japanese *Saxifraga* species (*S. stolonifera* [*Sst*], *S. fortune* [*Sf*], *S. nipponica* [*Sn*], *S. cortusifolia* [*Sc*], and *S. rebunshirensis* [*Sr*]) were performed using reverse-phase column chromatography to identify their major virucidal components. The components of each dried plant were extracted with acetone-H_2_O (4:1), and this rough extract was applied to a Diaion HP-20 open column to obtain methanol (MeOH)-H_2_O (2:3)- and MeOH-H_2_O (3:2)-eluted fractions (*Sst*-1C, *Sst*-1D, *Sf*-1C, *Sf*-1D, *Sn*-1C, *Sn*-1D, *Sc*-1C, *Sc*-1D, and *Sr*-1C). The fractions were then subjected to reverse-phase high-performance liquid chromatography (HPLC) to produce fractions containing condensed tannins (*Sst-*2R, *Sst-*3H+I, *Sf-*2H, *Sf-*3G, *Sn*-2E+3F, *Sc*-2E+3F, and *Sr*-2E). The *Sst*-2R fraction was determined to contain condensed tannins (proanthocyanidins) using nuclear magnetic resonance (NMR) and mass spectrometry (MS). In general, the ^13^C NMR spectra of condensed tannins reveal a substantially greater amount of information about skeletons and substituents than ^1^H NMR spectra. In this study, the ^13^C NMR spectrum of *Sst*-2R (Fig. 1) showed features of condensed tannins (16). In the aromatic field, resonances assigned to flavonoid units (δ_C_ 158–153 [C-5, 7, 8a], 132–129 [C-1′], 146–143 [C-3′, 4′], 122–118 [C-6′], 117–113 [C-2′, 5′], 108–105 [C-8], 98–94 [C-4a, 6]) were observed. These resonances as well as those in the aliphatic region at δ_C_ 80–72 (C-2, 3) and 35–32 (C-4) suggested that the unit skeleton was flavan-3-ol. The C-2 carbons of epicatechin-3-*O*-gallate and epicatechin were shielded compared to those of catechin-3-*O*-gallate and catechin (17, 18), suggesting that the flavan-3-ol was epicatechin. Although further investigation will be needed because the ^13^C NMR peaks of *Sst*-2R were broad, the chemical shift values of C-2, C-3, and C-4 were close to those of (2α,3α,4β)-4-arylflavan-3ol (19) and epicatechin-(4β→8)-epicatechin-type oligomers (20). The carbonyl resonances at δ_C_ 167–164 (C-7′′) and aliphatic resonances at δ_C_ 146–143 ppm (C-3′′, 5′′), 140–137 (C-4′′), 122–118 (C-1′′), and 111–109 (C-2′′, 6′′) showed the presence of galloyl moieties. These data show that *Sst*-2R contains repeated structures of epicatechin-3-*O*-gallate-(4β→8)-epicatechin-3-*O*-gallate, a finding which is supported by previous reports of SS-tannin 1 and related compounds derived from *S. stolonifera* (15, 21).

**FIG 2:**
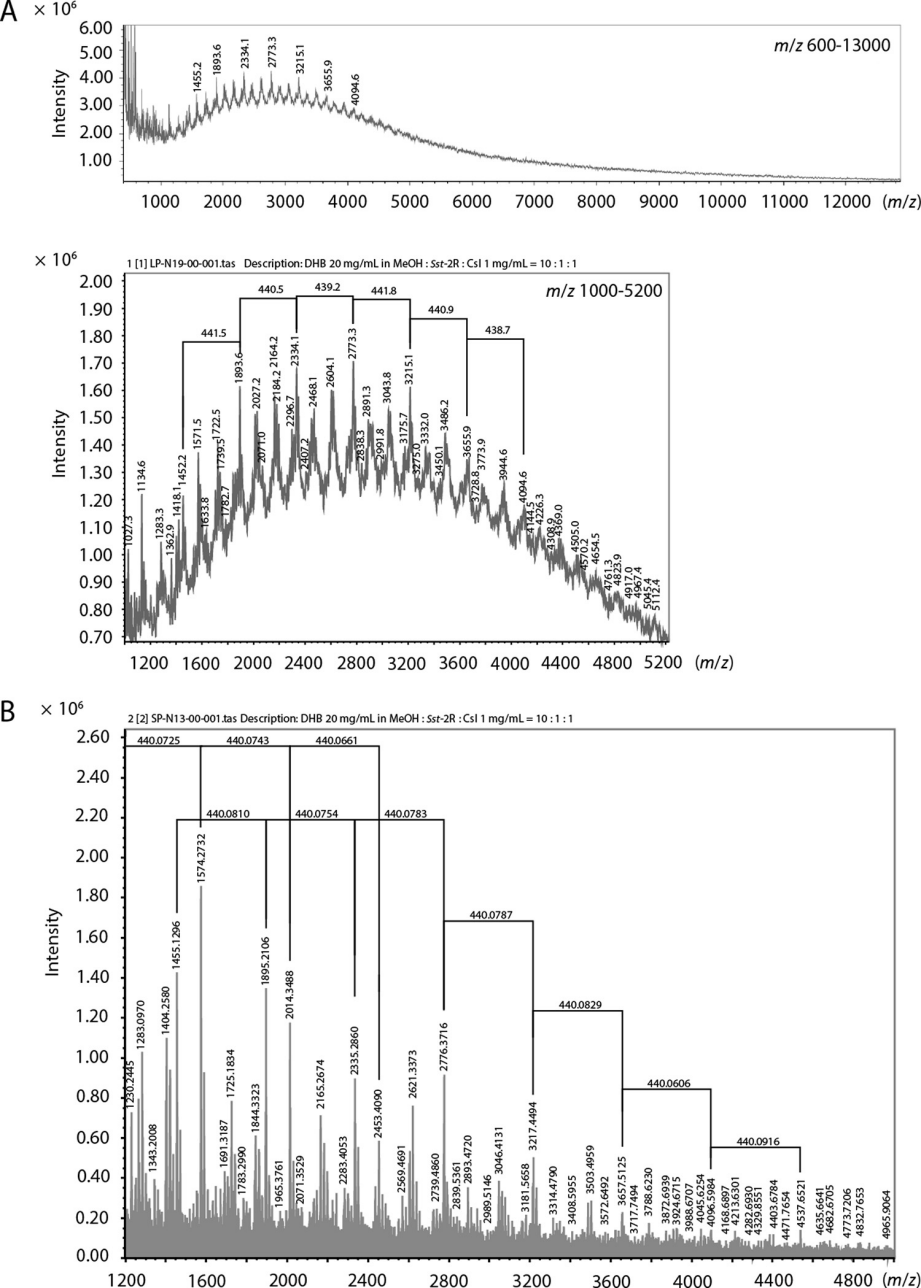
TOF-MS spectra of *Sst*-2R. Linear (A) and spiral (B) modes.

**FIG 3:**
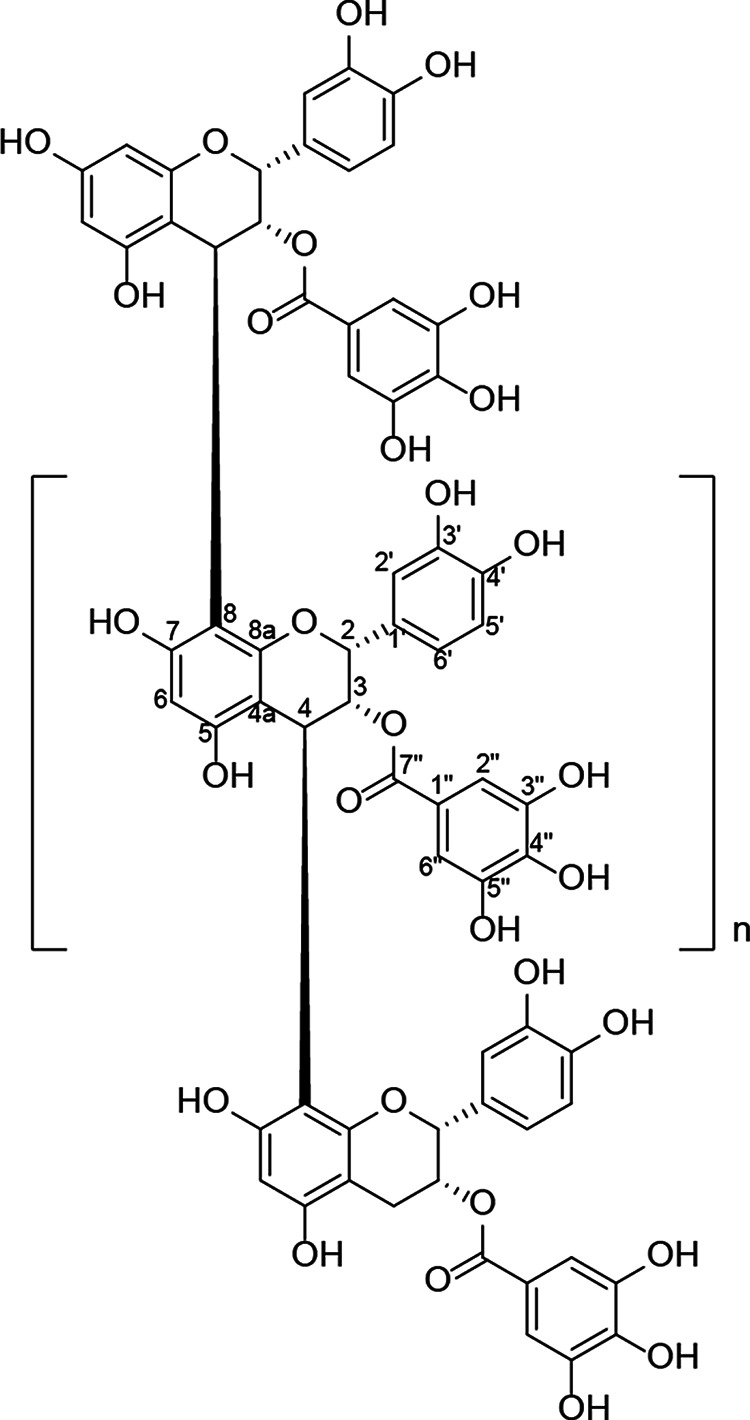
Expected chemical structures of *Saxifraga* species-derived condensed tannins.

In the linear mode time-of-flight MS (TOF-MS) data of *Sst*-2R, ion peaks of *m/z* 8,000 were observed, and prominent ion peaks were observed at 440-*m/z* intervals (Fig. 2A). The spiral mode TOF-MS data of *Sst*-2R showed the details of the ion peaks, which supported the 440-*m/z* intervals (Fig. 2B). The main peaks in the TOF-MS spectra obtained using cesium iodide were confirmed as originating from cesium adducts of oligomer molecules by comparison with the data obtained using a different cationization agent (sodium iodide) (see Fig. S1 in the supplemental material). The 440-*m/z* interval peaks supported the hypothesis that the unit of the oligomer was epicatechin-3-*O*-gallate (C_22_H_16_O_10_). Although the peak picking data in the experimental MS spectra showed some deviations due to molecular isotopes, they corresponded to the cesium adduct ions at around *m/z* 1,455 [C_66_H_50_O_30_+Cs]^+^, *m/z* 1,895 [C_88_H_66_O_40_+Cs]^+^, *m/z* 2,335 [C_110_H_82_O_50_+Cs]^+^, *m/z* 2,776 [C_132_H_98_O_60_+Cs]^+^, *m/z* 3,217 [C_154_H_114_O_70_+Cs]^+^, *m/z* 3,658 [C_176_H_130_O_80_+Cs]^+^, *m/z* 4,097 [C_198_H_146_O_90_+Cs]^+^, and *m/z* 4,538 [C_220_H_162_O_100_+Cs]^+^, which showed the presence of oligomers with 3 to 10 degrees of oligomerization (Fig. 3). The presence of 18-degree oligomers was expected by the signals until *m/z* 8,000 in the linear mode TOF-MS. The TOF-MS experimental procedures and data in this study clearly showed the structural features and oligomerization degrees of condensed tannins. Based on these data, *Sst*-2R was determined to be the purified condensed tannin fraction. The chemical structures of the *Saxifraga* species-derived condensed tannins are shown in Fig. 3.

The TOF-MS spectra of *Sf*-3G were similar to those of *Sst-*2R (Fig. S2), suggesting the presence of condensed tannins in *S*. *fortunei*. The ^13^C NMR spectroscopic features of fractions *Sst*-3H+I, *Sf-*2H, *Sf-*3G, *Sn*-2E+3F, *Sc*-2E+3F, and *Sr*-2E (Fig. S3) were also similar to those of *Sst*-2R, and these fractions were therefore also considered purified condensed tannin fractions.

### Virucidal activities of *Saxifraga* species-derived samples.

The virucidal activities of 100 μg/mL of five Japanese *Saxifraga* species-derived rough extracts were evaluated using a reaction time of 1 min. The *Sst*-, *Sf*-, *Sr*-, and *Sc*-derived rough extracts induced reduction of >1 log_10_ 50% tissue culture infective doses (TCID_50_)/mL in the viral titers of the SARS-CoV-2 ancestral strain, IAV, and FCV. In addition, the *Sr*-derived rough extract induced a 1-log_10_-TCID_50_/mL reduction in the viral titer of MNV (Table S1). Because the Mongolian *S. spinulosa*-derived 1C and 1D fractions showed potent virucidal activity in our previous study (11), the activities of five Japanese *Saxifraga* species-derived 1C and 1D fractions were also evaluated. SARS-CoV-2, IAV, and FCV were treated with 25-μg/mL samples for 10 s, while MNV was treated with 100-μg/mL samples for 1 min. In this experimental setting, although the degrees of reduction in viral titers differed among the 1C and 1D groups of fractions derived from different species, almost all of these samples showed statistically significant virucidal activities against four virus species. The virucidal activities of the *Sst*-derived fractions 2A to 2T were also evaluated. While *Sst*-2A to *Sst-*2M showed no or weak virucidal activity, *Sst*-2N to *Sst*-2T showed more potent virucidal activity, with *Sst*-2R exhibiting the strongest activity. Reductions of ≥3.13, ≥3.00, and 2.50 log_10_ TCID_50_/mL in the viral titers of the SARS-CoV-2 ancestral strain, IAV, and FCV, respectively, were induced by 25 μg/mL *Sst*-2R within 10 s. A 1.75-log_10_-TCID_50_/mL reduction in the viral titer of MNV was induced by 100 μg/mL *Sst*-2R in 1 min. Because *Sst*-2R was the purified condensed tannin fraction, the equivalent purified condensed tannin fractions derived from other *Saxifraga* species were also tested. All four samples—*Sf*-2H, *Sf*-3G, *Sn*-2E+3F, and *Sc*-2E+3F—showed virucidal activities comparable to that of *Sst*-2R. In general, the activities of these purified condensed tannin fractions were comparable to or stronger than that of each *Saxifraga* species-derived 1C or 1D fraction (Table 1).

**TABLE 1:** Virucidal activities of *Saxifraga* species-derived fractions against multiple virus species

Sample	Reduction in titer (log_10_ TCID_50_/mL) of target virus^*a*^			
	SARS-CoV-2^*b*^^,^^*c*^	IAV^*c*^	FCV^*c*^	MNV^*d*^
*Sst*-1C	≥4.00 ± 0.00***	≥2.50 ± 0.58**	1.63 ± 0.48**	1.25 ± 0.50*
*Sst*-1D	≥3.50 ± 0.50**	≥1.88 ± 0.75*	1.75 ± 0.29**	1.50 ± 0.71*
*Sf*-1C	≥3.56 ± 0.38***	1.25 ± 0.65*	1.63 ± 0.63*	1.13 ± 0.48*
*Sf*-1D	≥3.81 ± 0.13***	≥1.88 ± 0.48**	1.75 ± 0.65*	1.63 ± 0.48**
*Sr*-1C	≥3.75 ± 0.50***	≥1.88 ± 0.48**	2.00 ± 0.50*	1.00 ± 0.00***
*Sr*-1D	1.50 ± 0.91*	0.92 ± 0.54**	2.00 ± 0.50*	0.50 ± 0.54*
*Sn*-1C	1.88 ± 0.63**	0.50 ± 0.29**	1.38 ± 0.85*	0.44 ± 0.42*
*Sn*-1D	2.38 ± 0.63**	−0.13 ± 0.25^ns^	1.50 ± 0.41**	0.44 ± 0.42*
*Sc*-1C	≥3.56 ± 0.38***	≥2.00 ± 0.71*	1.50 ± 0.50*	1.38 ± 0.85*
*Sc*-1D	≥3.50 ± 0.68**	1.75 ± 0.29**	1.67 ± 0.29**	0.69 ± 0.92^ns^
*Sst*-2A	0.25 ± 0.29^ns^	0.00 ± 0.00^ns^	0.33 ± 0.29^ns^	0.33 ± 0.76^ns^
*Sst*-2B	−0.13 ± 0.25^ns^	−0.50 ± 0.50^ns^	0.33 ± 0.29^ns^	0.33 ± 0.76^ns^
*Sst*-2C	0.00 ± 0.41^ns^	0.33 ± 0.58^ns^	0.67 ± 1.04^ns^	0.33 ± 0.76^ns^
*Sst*-2D	0.13 ± 0.63^ns^	0.17 ± 0.76^ns^	0.50 ± 0.87^ns^	0.17 ± 1.04^ns^
*Sst*-2E	0.25 ± 0.29^ns^	0.00 ± 1.00^ns^	0.83 ± 0.76^ns^	0.00 ± 0.87^ns^
*Sst*-2F	−0.13 ± 0.25^ns^	−0.33 ± 0.76^ns^	0.67 ± 1.04^ns^	0.33 ± 0.76^ns^
*Sst*-2G	0.63 ± 0.25*	0.00 ± 0.87^ns^	0.33 ± 0.58^ns^	0.50 ± 0.50^ns^
*Sst*-2H	0.50 ± 0.41^ns^	0.17 ± 0.76^ns^	0.33 ± 0.58^ns^	−0.17 ± 0.29^ns^
*Sst*-2I	0.38 ± 0.25^ns^	−0.83 ± 0.29^ns^	0.67 ± 0.29^ns^	0.00 ± 0.50^ns^
*Sst*-2J	0.50 ± 0.58^ns^	−0.50 ± 0.50^ns^	0.50 ± 0.50^ns^	0.33 ± 0.76^ns^
*Sst*-2K	0.63 ± 0.75^ns^	0.25 ± 0.50^ns^	0.13 ± 0.48^ns^	−0.38 ± 0.63^ns^
*Sst*-2L	1.25 ± 0.29**	0.13 ± 0.63^ns^	0.50 ± 0.58^ns^	0.00 ± 0.41^ns^
*Sst*-2M	1.50 ± 0.41**	0.63 ± 0.48^ns^	1.93 ± 0.67***	0.38 ± 0.25^ns^
*Sst*-2N	2.50 ± 0.41**	2.00 ± 0.41**	2.29 ± 0.76***	1.50 ± 0.41**
*Sst*-2O	2.13 ± 0.63**	1.88 ± 0.63**	2.29 ± 0.86***	1.25 ± 0.29**
*Sst*-2P	2.13 ± 0.48**	1.13 ± 0.63*	1.50 ± 0.82*	0.38 ± 0.85^ns^
*Sst*-2Q	2.25 ± 0.29***	≥2.88 ± 0.25***	2.14 ± 0.63***	2.00 ± 0.71*
*Sst*-2R	≥3.13 ± 0.25***	≥3.00 ± 0.00***	2.50 ± 0.82***	1.75 ± 0.29**
*Sst*-2S	2.25 ± 0.50***	≥2.50 ± 0.71**	1.86 ± 0.80***	1.88 ± 0.25***
*Sst*-2T	2.00 ± 0.41***	≥2.38 ± 0.48**	1.88 ± 0.95*	1.00 ± 0.71^ns^
*Sf*-2H	≥2.88 ± 0.25***	≥2.63 ± 0.25***	2.38 ± 0.75**	2.00 ± 0.58**
*Sf*-3G	≥3.00 ± 0.41***	≥2.63 ± 0.25***	2.00 ± 0.71*	1.88 ± 0.48**
*Sn*-2E+3F	≥2.88 ± 0.25***	≥2.63 ± 0.25***	2.13 ± 1.11*	1.63 ± 0.63*
*Sc*-2E+3F	≥2.75 ± 0.29***	≥2.63 ± 0.25***	2.00 ± 0.71*	1.38 ± 0.63*

a Calculated as (viral titer of DMSO group) − (viral titer of sample group). Values are means and standard deviations (SD). The degree of the reduction in the viral titer represents the degree of virucidal activity of each test sample. Statistical analysis was done with Student’s *t* test (*n* = 4 to 8). *, *P *< 0.05; **, *P *< 0.01; ***, *P *< 0.001; ns, not significant. Gray shading highlights values with no significant differences.

b Ancestral strain.

c Sample concentration was 25 μg/mL; reaction time was 10 s.

d Sample concentration was 100 μg/mL; reaction time was 1 min.

To evaluate whether the virucidal activity of *Sst*-2R is concentration and time dependent, the virucidal activity of *Sst*-2R at various concentrations against SARS-CoV-2, as a representative enveloped virus, and MNV, as a representative nonenveloped virus, was evaluated at various reaction times. The results showed that the degree of SARS-CoV-2 and MNV inactivation by *Sst*-2R was greater at higher concentrations and longer reaction times (Table S2).

The virucidal activities of *Sst*-1C, *Sst*-1D, and *Sst*-2R against multiple variant strains (Alpha, Beta, Gamma, Delta, and Omicron strains) of SARS-CoV-2 were evaluated. The 25-μg/mL fractions induced a ≥2.69-log_10_-TCID_50_/mL reduction in the viral titers of all the tested variants within 10 s (Table 2).

**TABLE 2:** Virucidal activities of *Saxifraga* species-derived fractions against SARS-CoV-2 variant strains

Sample	Reduction in titer (log_10_ TCID_50_/mL) of target SARS-CoV-2 strain^*a*^				
	Alpha	Beta	Gamma	Delta	Omicron
*Sst*-1C	≥3.00 ± 0.71***	≥3.63 ± 0.48***	≥4.63 ± 0.48***	≥3.75 ± 0.65**	≥2.81 ± 0.60***
*Sst*-1D	≥3.50 ± 0.54***	≥3.88 ± 0.48***	≥4.38 ± 0.48***	≥3.50 ± 0.58**	≥2.69 ± 0.53***
*Sst*-2R	≥3.88 ± 0.44***	≥4.00 ± 0.00***	≥4.63 ± 0.25***	≥3.63 ± 0.25***	≥3.56 ± 0.42***

a Sample concentration was 25 μg/mL; reaction time was 10 s. Reduction in viral titer was calculated as (viral titer of DMSO group) − (viral titer of sample group). Values are means and SD. The degree of the reduction in the viral titer represents the degree of virucidal activity of each test sample. Statistical analysis was done with Student’s *t* test (*n *= 4 to 8). **, *P *< 0.01; ***, *P *< 0.001.

### Virucidal activities of 70% ethanol, 2,000 ppm sodium hypochlorite solution, and catechin derivatives.

The virucidal activities of currently available disinfectants—ethanol and sodium hypochlorite solution—were also evaluated to compare them to those of *Saxifraga* species-derived fractions. The tested concentrations of ethanol and sodium hypochlorite solution were 70% and 2,000 ppm, respectively. Such concentrations are adopted for inactivation of viruses in the real world (Centers for Disease Control and Prevention [CDC]: https://www.cdc.gov/infectioncontrol/pdf/guidelines/disinfection-guidelines-H.pdf). The CDC recommends the use of 1,000 to 5,000 ppm of sodium hypochlorite solution for the inactivation of norovirus on environmental surfaces (https://www.cdc.gov/mmwr/preview/mmwrhtml/rr6003a1.htm).

In general, nonenveloped viruses are resistant to ethanol. However, in the family *Caliciviridae*, the susceptibility to ethanol differs among virus species and strains, and certain viruses are partially or effectively inactivated by ethanol (22–24). The virucidal activities of ethanol and sodium hypochlorite solution against four pathogenic viruses were analyzed under the experimental conditions used for the *Saxifraga* species-derived fractions. The results showed that 70% ethanol and 2,000 ppm sodium hypochlorite solution exhibited more potent virucidal activities against four pathogenic viruses than *Saxifraga* species-derived fractions at the concentrations in Table 1 (Table S3).

The virucidal activities of catechin derivatives, which are known to have virucidal activity against a wide range of virus species (11, 25, 26), were also evaluated as representatives of naturally derived virucidal compounds. The virucidal activities of five catechin derivatives were analyzed with the concentrations and experimental conditions used for *Saxifraga* species-derived fractions. The results showed that the five catechin derivatives did not show any virucidal activities (Table S4).

### Cytotoxicity of *Saxifraga* species-derived fractions, ethanol, and sodium hypochlorite solution.

Next, we compared the cytotoxicities of *Sst*-1C, *Sst*-1D, *Sst*-2R, ethanol, and sodium hypochlorite solution against four cell lines: Vero E6/transmembrane protease serine 2 (TMPRSS2), Madin-Darby canine kidney (MDCK), Crandell-Rees feline kidney (CRFK), and RAW264 cells. The Vero E6/TMPRSS2, MDCK, CRFK, and RAW264 cells were used for virucidal tests against SARS-CoV-2, IAV, FCV, and MNV, respectively. The 50% cytotoxic concentrations (CC_50_) against the four cell lines calculated in the cytotoxicity test were found to be lower than the concentrations that showed virucidal activities in the virucidal tests (Table 1 and Table S3) for all test samples. When Vero E6/TMPRSS2, MDCK, and CRFK cells were targeted, the difference between the concentration that showed virucidal activity in the virucidal test and the CC_50_ (calculated as concentration showing virucidal activity in virucidal test/CC_50_ in cytotoxicity test) was markedly smaller in the case of *Sst*-1C, *Sst*-1D, and *Sst*-2R than in the case of ethanol and sodium hypochlorite solution. This result indicates that the concentrations of ethanol and sodium hypochlorite used in the virucidal tests (70% and 2,000 ppm, respectively) were markedly higher than the CC_50_, while those of *Sst*-1C, *Sst*-1D, and *Sst*-2R (25 μg/mL) were higher than the CC_50_ but remained relatively close. When RAW264 cells were targeted, the difference between the concentration that showed virucidal activity in the virucidal test and the CC_50_ was comparable among all test samples (Table S5).

### Virucidal activities of *Sst*-1D-containing creams.

We evaluated the virucidal activities of 5% and 10% *Sst*-1D-containing creams. When viral solutions were covered with films to which cream had been applied, *Sst*-1D-containing creams induced ≥2.38-, ≥3.00-, ≥2.63-, and ≥1.88-log_10_-TCID_50_/mL reductions in the viral titers of the SARS-CoV-2 ancestral strain, IAV, FCV, and MNV, respectively, within 10 min (Table 3).

**TABLE 3:** Virucidal activity of *Sst*-1D-containing cream against multiple virus species^*a*^

Sample cream	Reduction in titer (log_10_ TCID_50_/mL) of target virus^*a*^			
	SARS-CoV-2^*b*^	IAV	FCV	MNV
5% *Sst*-1D	≥2.38 ± 1.11	≥3.13 ± 0.48	≥2.75 ± 0.65	≥1.88 ± 0.48
10% *Sst*-1D	≥2.75 ± 0.60	≥3.00 ± 0.60	≥2.63 ± 0.48	≥2.00 ± 0.71

a Reaction time was 10 min. Reduction in viral titer is calculated as (viral titer of base cream group) − (viral titer of sample cream group). Values are means and SD. The degree of the reduction in the viral titer represents the degree of virucidal activity of each test sample. Statistical analysis was done with Student’s *t* test (*n *= 4 to 8). *P *< 0.001 for all values.

b Ancestral strain.

### Impact of *Saxifraga* species-derived condensed tannins on viral structural proteins.

**FIG 4:**
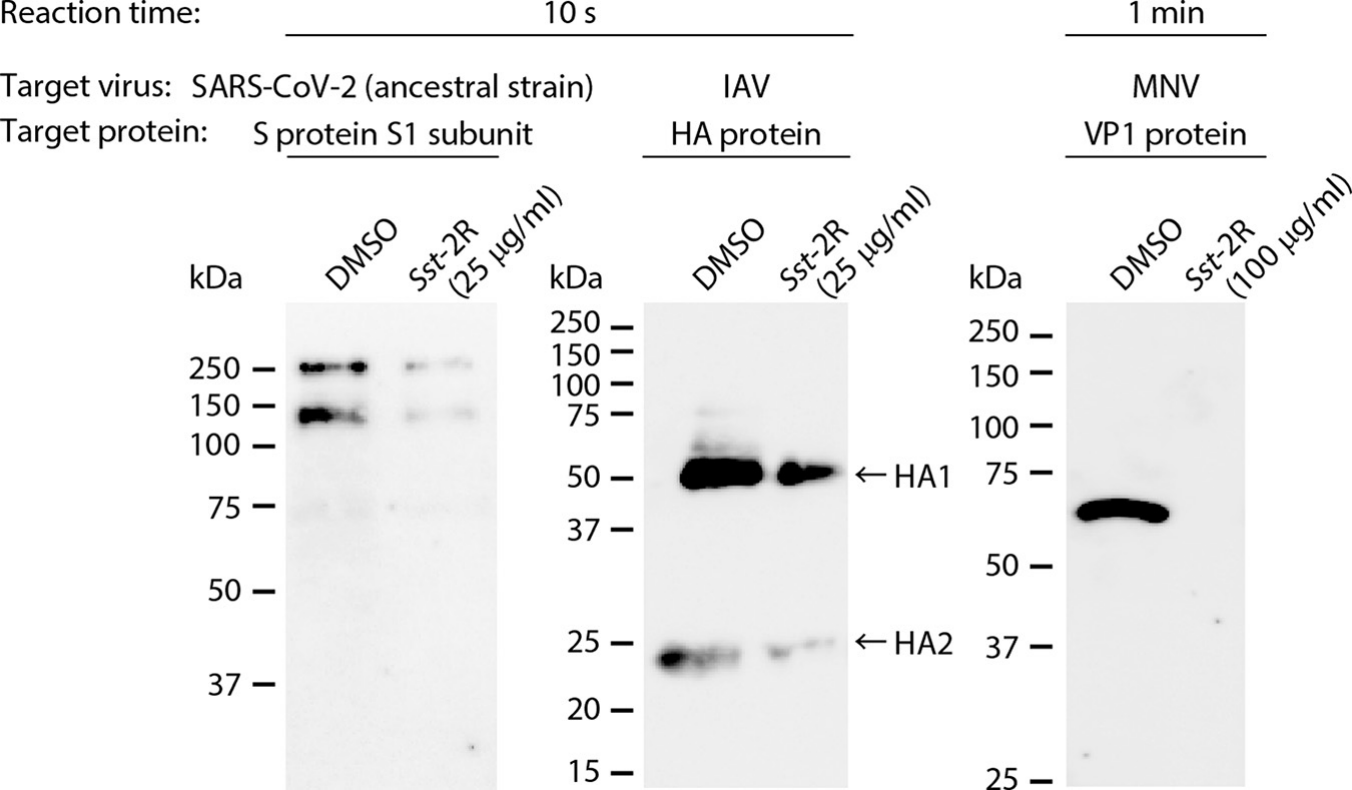
Effect of *Sst*-2R on viral structural proteins. SARS-CoV-2 ancestral strain, IAV, or MNV solution was mixed with DMSO or *Sst*-2R. The concentration of *Sst*-2R in the mixture was 25 μg/mL (against SARS-CoV-2 or IAV) or 100 μg/mL (against MNV). After 10 s (against SARS-CoV-2 or IAV) or 1 min (against MNV), Western blotting targeting each viral structural protein was visualized.

The expression patterns of viral structural proteins in dimethyl sulfoxide (DMSO)- or *Sst*-2R-treated viruses were analyzed using Western blotting. After 10 s of reaction time, the intensities of bands of the SARS-CoV-2 spike (S) protein S1 subunit and the IAV hemagglutinin (HA) protein HA1 and HA2 subunits were reduced in viruses treated with 25 μg/mL *Sst*-2R compared to DMSO-treated ones. After 1 min of reaction time, a clear specific band of the MNV VP1 protein was observed in DMSO-treated viruses, while the band was not present in those treated with 100 μg/mL *Sst*-2R (Fig. 4). The effect of *Sst*-2R treatment on nonviral proteins was also evaluated. Bovine serum albumin (BSA) was used as a representative nonviral protein. The intensity of the BSA band was reduced after treatment with 100 μg/mL *Sst*-2R compared to DMSO and 25 μg/mL *Sst*-2R treatments after 10 s of reaction time (Fig. S4).

### Morphology of viral particles treated with condensed tannins derived from *Saxifraga* species.

**FIG 5:**
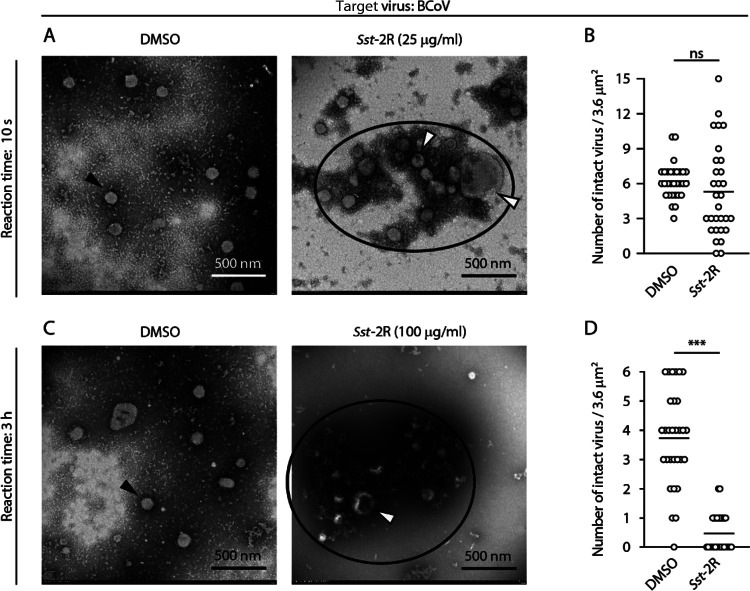
Morphology of *Sst*-2R-treated BCoV particles. BCoV solution was mixed with DMSO or *Sst*-2R. The concentration of *Sst*-2R in the mixture was 25 μg/mL (A and B) or 100 μg/mL (C and D). After 10 s (A and B) or 3 h (C and D), the viral particles were observed using TEM. TEM images of BCoV are shown (A and C). Black arrowheads, viral particles with normal structure; white arrowheads, viral particles with abnormal structure; black outlines, aggregated viral particles. The numbers of intact viral particles in one field of view were counted (B and D). Student’s *t* test was done (*n *= 30 fields). ***, *P < *0.001; ns, not significant.

**FIG 6:**
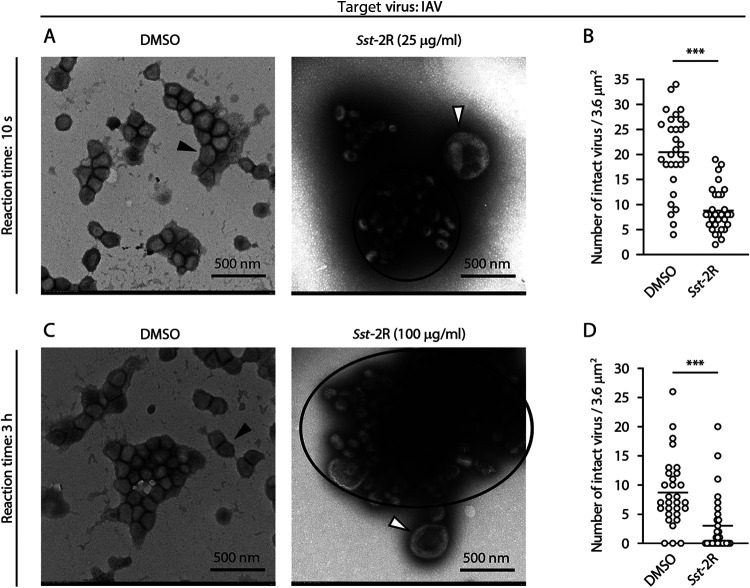
Morphology of *Sst*-2R-treated IAV particles. IAV solution was mixed with DMSO or *Sst*-2R. The concentration of *Sst*-2R in the mixture was 25 μg/mL (A and B) or 100 μg/mL (C and D). After 10 s (A and B) or 3 h (C and D), the viral particles were observed using TEM. TEM images of IAV are shown (A and C). Black arrowheads, viral particles with normal structure; white arrowheads, viral particles with abnormal structure; black outlines, aggregated viral particles. The numbers of intact viral particles in one field of view were counted (B and D). Student’s *t* test was done (*n *= 30 fields). ***, *P < *0.001.

**FIG 7:**
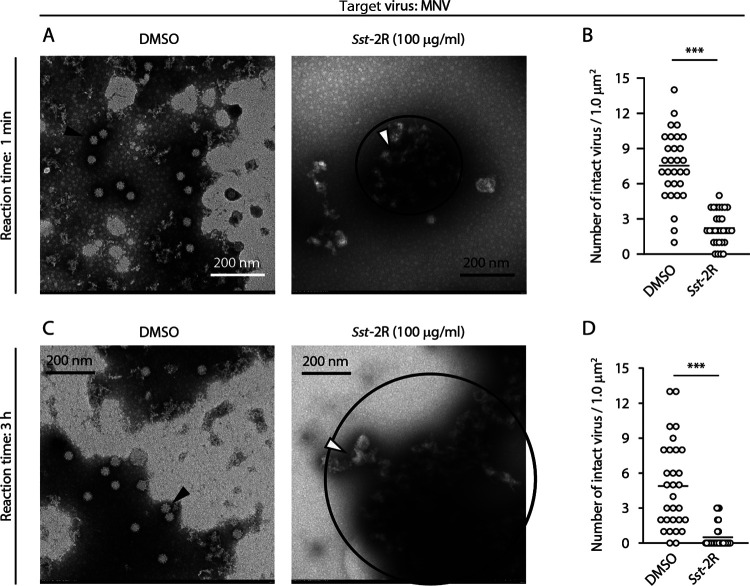
Morphology of *Sst*-2R-treated MNV particles. MNV solution was mixed with DMSO or *Sst*-2R. The concentration of *Sst*-2R in the mixture was 100 μg/mL. After 1 min (A and B) or 3 h (C and D), the viral particles were observed using TEM. TEM images of MNV are shown (A and C). Black arrowheads, viral particles with normal structure; white arrowheads, viral particles with abnormal structure; black outlines, aggregated viral particles. The numbers of intact viral particles in one field of view were counted (B and D). Student’s *t* test was done (*n *= 30 fields). ***, *P < *0.001.

DMSO- or *Sst*-2R-treated bovine coronavirus (BCoV: surrogate virus for SARS-CoV-2), IAV, and MNV particles were directly observed using transmission electron microscopy (TEM). Following DMSO treatment, many BCoV particles had clear spike proteins and intact envelopes after 10 s of reaction time. The size of these viral particles appeared to be around 100 nm, the normal size. Following treatment with 25 μg/mL *Sst*-2R, some abnormal viral particles were observed that were larger than normal and had envelopes that appeared to be disrupted. Aggregation of viral particles was also observed following *Sst*-2R treatment (Fig. 5A and Fig. S5A). However, there was no significant difference in the number of viral particles with normal size and clear spike proteins and envelopes, defined as intact viruses, between the DMSO and *Sst*-2R treatments (Fig. 5B). The effect of *Sst*-2R at a higher concentration and longer reaction time on BCoV particles was also evaluated. The number of intact viral particles was reduced by treatment with 100 μg/mL *Sst*-2R in a 3-h reaction time (Fig. 5C and D and Fig. S5B). Following DMSO treatment, many intact IAV particles that had clear spike proteins and intact envelopes remained in both the 10-s and 3-h reaction times. Following both 25 μg/mL and 100 μg/mL *Sst*-2R treatments (reaction times, 10 s and 3 h, respectively), many abnormal viral particles were observed that were larger than normal and had envelopes that appeared to be disrupted. Aggregation of viral particles was also observed following *Sst*-2R treatment. The degree of aggregation by 100 μg/mL *Sst*-2R in a 3-h reaction time appeared to be more potent than that by 25 μg/mL *Sst*-2R in a 10-s reaction time (Fig. 6A and C; Fig. S6A and B). The number of intact viral particles was reduced by *Sst*-2R treatment regardless of its concentration and reaction time (Fig. 6B and D). Many MNV particles with normal capsids were observed following DMSO treatment at both the 1-min and 3-h reaction times. However, following 100-μg/mL *Sst*-2R treatment at the reaction times of 1 min and 3 h, the number of intact viruses decreased, and many viral particles showed damaged capsid structures (Fig. 7A to D). Aggregation of viral particles was also observed following *Sst*-2R treatment. The degree of aggregation by 100 μg/mL *Sst*-2R in 3-h reaction time appeared to be more potent than that in the 1-min reaction time (Fig. 7A and C; Fig. S7A and B).

## DISCUSSION

In this study, we showed that almost all of the five 1C and 1D fractions derived from Japanese *Saxifraga* species exhibited rapid and potent virucidal activities against both enveloped and nonenveloped virus species. The virucidal activities of these fractions were stronger than those of the rough extracts (Table 1 and Table S1). Using activity-guided separation, we identified condensed tannins as the active chemical constituents that play a central role in the strong virucidal activity of these *Saxifraga*-derived fractions (Table 1). The *Sst*-2R fraction from *S*. *stolonifera* was a typical fraction containing abundant *Saxifraga* species-derived condensed tannins. ^13^C NMR and TOF-MS spectral analyses led to the conclusion that the *Saxifraga* species-derived tannins were mixtures of oligomers of epicatechin-3-*O*-gallate (Fig. 3). Oligomers with a degree of oligomerization in the range of 3 to 10 were established as main components of the fraction, and TOF-MS data suggested the presence of around 18-degree oligomers (Fig. 2). The five Japanese species of *Saxifraga* plants used in this study contained condensed tannins in the 1C and 1D fractions. Although the activities of the rough extract and the initial fractions of *S*. *nipponica* (*Sn*-rough extract, *Sn*-1C, and *Sn*-1D) were relatively weak, the purified condensed tannin fraction from this plant (*Sn*-2E+3F) showed potent activity. The differences in virucidal activity levels between plant species may be explained by the concentration of condensed tannins in the fractions. Consistent with this hypothesis, the virucidal activity of *Sst*-2R was concentration dependent as well as time dependent (Table S2).

The virucidal activities of *Saxifraga-*derived samples at concentrations of 25 μg/mL (against SARS-CoV-2, IAV, and FCV) and 100 μg/mL (against MNV) were weaker than those of 70% ethanol and 2,000 ppm sodium hypochlorite solution (Table 1 and Table S3). Nevertheless, when the Vero E6/TMPRSS2, MDCK, and CRFK cells were targeted, the difference between the concentration showing virucidal activity in the virucidal test (Table 1; Table S3) and the CC_50_ was markedly smaller in the case of *Sst*-1C, *Sst*-1D, and *Sst*-2R than in the case of ethanol and sodium hypochlorite solution (Table S5). The ability of *Saxifraga* species-derived samples to achieve rapid virus inactivation at a relatively low toxic concentration compared to the currently available disinfectants could be advantageous for practical use. Although the actual toxicity to the living body should be evaluated using multiple *in vivo* toxicity tests, including skin irritation tests, our *in vitro* cytotoxicity tests against various cell lines suggest that the *Saxifraga* species-derived samples could be applied in situations where ethanol and sodium hypochlorite solution cannot be used due to their cytotoxicity. When *Saxifraga* species-derived samples are added to ethanol- or sodium hypochlorite-based disinfectants, they may contribute to reducing the adverse reactions caused by these disinfectants by reducing the contained amount of ethanol or sodium hypochlorite needed. Because some people are intolerant to alcohol and sodium hypochlorite cannot be applied to the skin, the use of *Saxifraga* species-derived samples could compensate for the limitations of the currently available disinfectants. One limitation of the present study is that the virucidal activities of 2,000 ppm sodium hypochlorite solution and *Saxifraga* species-derived samples were compared against FCV and MNV in suspension. However, the CDC-recommended high concentration (1,000 to 5,000 ppm) of sodium hypochlorite solution is for the inactivation of HuNoV on contact surfaces. Therefore, future analysis comparing the virucidal activities of 2,000 ppm sodium hypochlorite solution and *Saxifraga* species-derived samples should be performed against FCV and MNV on surfaces to obtain information that is more significant for the practical application of these samples.

The virucidal activities of catechin derivatives, particularly epigallocatechin-3-*O*-gallate, have been demonstrated in many previous studies (11, 25, 26). In our previous study, gallocatechin-3-*O*-gallate and epigallocatechin-3-*O*-gallate at a concentration of 25 μg/mL induced 1.7- and 1.0-log_10_-TCID_50_/mL, 2.17- and 1.17-log_10_-TCID_50_/mL, and ≥2.5- and ≥2.5-log_10_-TCID_50_/mL reductions in the viral titers of SARS-CoV-2, IAV, and FCV, respectively, within 1 min. However, neither of these catechin derivatives showed virucidal activity against MNV within 10 min at a concentration of 100 μg/mL (11). These previous results and our current results indicate that the activity of *Saxifraga* species-derived condensed tannins was considerably stronger than that of catechin derivatives (Table 1 and Table S4).

Tannins are high-molecular-weight polyphenolic compounds that are found in several types of plants. Tannins have biological activities such as antioxidant, antitumor, astringent, and broad-range antimicrobial properties, which contribute to the protection of plants against oxidative stress and infection by pathogens. Tannins are classified into two main types: condensed tannins and hydrolysable tannins (27, 28). Both condensed and hydrolysable tannins have antiviral activity. The inhibitory activities of tannins, including the epicatechin-3-*O*-gallate trimer and tetramer, against herpes simplex virus have been investigated (29). Ueda et al. (30) evaluated the virucidal activities of multiple plant extracts containing tannins against a broad range of virus species, including IAV, FCV, and MNV. The results showed that a 0.25% persimmon extract containing condensed tannin induced a ≥4-log_10_-TCID_50_/mL reduction in the viral titer of all 12 of the tested virus species within 3 min. Furthermore, the viral titer of IAV treated with 0.25% persimmon extract decreased below the detection limit within 30 s. In their study, it was found that the persimmon extract blocked the hemagglutination activity of IAV (30). Such a phenomenon was also observed in IAV treated with *S. spinulosa*-derived 1C and 1D fractions in our previous study (11). In addition, *S. spinulosa*-derived 1C and 1D fractions were found to inhibit the neuraminidase activity of IAV (11). Ueda et al. (30) showed that in sodium dodecyl sulfate-polyacrylamide gel electrophoresis (SDS-PAGE) analysis, the bands of IAV’s structural proteins disappeared from the original positions, and additional bands with high molecular masses appeared following treatment with persimmon extract. Similar results were observed in multiple viruses treated with *S. spinulosa*-derived 1C and 1D fractions (11). In the present study, although bands with high molecular masses were not detected, the clear disappearance or reduction of band intensity of multiple viral structural proteins was observed in Western blot analysis following *Sst*-2R treatment (Fig. 4). Our Western blot analysis results suggested that the antigen sites recognized by specific antibodies were destroyed or covered by *Saxifraga* species-derived condensed tannins, which may have resulted in the blockade of the binding of epitopes and antibodies. Because the intensity of the BSA band was also reduced by *Sst*-2R treatment (Fig. S4), *Saxifraga* species-derived condensed tannins seem to nonspecifically affect not only viral proteins but also nonviral proteins. TEM revealed that *Sst*-2R treatment induced morphological abnormalities and aggregation of viral particles, which occurred in all virus species. The reduction in the number of intact viral particles and the aggregation of viruses tended to be more pronounced when viruses were treated with *Sst*-2R at higher concentrations and longer reaction times (Fig. 5 to 7; Fig. S5 to S7). These results suggest that *Saxifraga* species-derived condensed tannins induce nonspecific aggregation and/or destruction of viral structural proteins, which results in the inhibition of viral protein functions. Haddad et al. (31) reported that tannic acid bound to the receptor-binding domain (RBD) of the S protein of SARS-CoV-2 and inhibited angiotensin-converting enzyme 2–RBD binding, which may contribute to blockage of the binding of SARS-CoV-2 to the host cell surface. Their *in silico* computational docking analysis demonstrated the possibility that tannic acid and RBD are bound by hydrogen bonds and hydrophobic interactions, and the pyrogallol structure seemed to play an important role in this binding. A study in which the inhibition of the hemagglutination and neuraminidase activities of IAV was compared among catechin derivatives showed that the 3-galloyl group in epicatechin-3-*O*-gallate and epigallocatechin-3-*O*-gallate plays an important role in this inhibition (32). Therefore, the abundant pyrogallol structures were presumed to contribute significantly to the potent virucidal activity of *Saxifraga* species-derived condensed tannins. TEM results suggested that *Saxifraga* species-derived condensed tannins destroyed not only viral structural proteins but also the envelope of BCoV and IAV (Fig. 5A and C and 6A and C). Many previous studies have shown that tannins interact with biological membranes and induce changes in their potential and permeability or destabilize their integrity (28, 33). Such functions may also have affected the viral envelope and contributed to the destruction of viral particles. Although *Saxifraga* species-derived condensed tannins inactivated both enveloped and nonenveloped viruses, the degree of inactivation by these samples seemed to be greater in enveloped viruses than in nonenveloped ones. Although *Saxifraga* species-derived condensed tannins affected the viral structural proteins, nonenveloped viruses may be more resistant than enveloped ones against these test samples as well as many other virucidal agents.

We also demonstrated the possible application of *Saxifraga* species-derived fractions as a virucidal cream, as did a previous study in which polyphenol-rich plant extract-containing hand cream exhibited SARS-CoV-2-inactivating activity (34). Phenolic compounds are regarded as safe, eco-friendly virucidal agents which can be used as an alternative to chemical disinfectants (35). Because tannins are contained in many edible plants and are empirically known to be safe for the living body (36), hand sanitizers and hand creams containing *Saxifraga* species-derived fractions may be valuable for hand hygiene. The potent and rapid virucidal activity of *Saxifraga* species-derived condensed tannins might also contribute to virus inactivation on the luminal surfaces of the respiratory and intestinal tracts, where local infection occurs, which might result in the blocking of viral infection of cells. Furukawa et al. (37) showed that orally administered persimmon-derived tannins attenuated the symptoms of SARS-CoV-2 infection and transmission of the virus. Some researchers have investigated the use of the Kampo medicine *maoto*, which contains tannin-rich ephedra herbs, to treat viral infections (38). Interestingly, the ephedra tannin, not the ephedrine alkaloids, was reported as a virucidal component in studies on ephedrine alkaloid-free ephedra herbs (38). In many countries, including Japan, traditional medicines to treat viral infections are gaining eminence, and tannins are studied as one of the active substances. *Saxifraga* species-derived fractions are a rich source of condensed tannins, which have unique properties that enable them to act against various viruses, as shown in this study. Furthermore, unlike ephedrine alkaloids, anthrones, and anthraquinones, no known *Saxifraga* ingredient exerts strong adverse effects in humans. These properties make *Saxifraga* extracts and their tannins promising antiviral candidates. Although the present study focused on the virucidal activity *in vitro*, the *in vivo* therapeutic effect of *Saxifraga* species-derived condensed tannins will be evaluated using a virus-infected animal model in future studies.

In conclusion, we identified the rapid and potent virucidal activity of *Saxifraga* species-derived condensed tannins. These tannins inactivated multiple virus species, both enveloped and nonenveloped, by inducing structural abnormalities in viral structural proteins and envelopes. In addition, these tannins inactivated multiple SARS-CoV-2 variant strains. This finding suggests that even if novel SARS-CoV-2 strains continue to arise, *Saxifraga* species-derived condensed tannins can exert nonspecific virucidal activity against these strains. Overall, our findings indicate that *Saxifraga* species-derived fractions containing condensed tannins can be used as virucidal agents against multiple pathogenic viruses on hands and environmental surfaces, thereby contributing to strict virus infection control measures.

## MATERIALS AND METHODS

### Plant materials.

*S. stolonifera* Curtis, *S. fortunei* Hook., *S. nipponica* Makino, *S. cortusifolia* Siebold & Zucc. (a synonym of *S. serotina* Sipliv.), and *S. rebunshirensis* Sipliv. (Saxifragaceae) were harvested in 2019 in Sendai, Japan. Toshihiro Murata identified the plant species, and the identifications were confirmed using their internal transcribed spacer 1 sequences (see the supplemental material). Voucher specimens (TMPUSst20200421, TMPUSf20201010, TMPUSn202010, TMPUSr20201010, and TMPUSc20201010) were deposited at the Herbarium of the Division of Pharmacognosy, Tohoku Medical and Pharmaceutical University (Sendai, Japan).

### *Saxifraga*-derived samples.

^13^C NMR (100 MHz) spectra were recorded using a JNM-AL400 FT-NMR spectrometer (JEOL, Ltd., Tokyo, Japan), and chemical shifts are provided as δ values with tetramethyl silane as the internal standard. Linear and spiral TOF-MS data were obtained using a JMS S3000 SpiralTOF Plus mass spectrometer (JEOL, Ltd.). A porous polymer gel (Diaion HP-20; Mitsubishi Chemical Co., Tokyo, Japan) was used for column chromatography. Preparative HPLC was performed using a JASCO 2089 instrument (columns: TSKgel octyldecyl silane [ODS]-120T, 21.5 by 300 mm; Tosoh, Tokyo, Japan) with UV detection at 210 nm.

The detailed extraction and fractionation procedures are described in the supplemental material. In brief, *S. stolonifera* (10 g), *S*. *fortunei* (61 g), *S*. *nipponica* (230 g), *S*. *cortusifolia* (19 g), and *S*. *rebunshirensis* (19 g) were extracted using acetone-H_2_O (4:1) at room temperature, and their rough extracts were obtained (*Sst*, 2.6 g; *Sf*, 10.9 g; *Sn*, 13.2 g; *Sc*, 3.0 g; and *Sr*, 0.77 g). Each extract was dissolved in H_2_O, and the solution was applied to a porous polymer gel (Diaion HP-20) open column and eluted with H_2_O-MeOH solvent systems. The following fractions were obtained: MeOH-H_2_O (2:3) (*Sst*-1C, 282 mg; *Sf*-1C, 362 mg; *Sn*-1C, 299 mg; *Sc*-1C, 344 mg; *Sr*-1C, 201 mg) and MeOH-H_2_O (3:2) (*Sst*-1D, 355 mg; *Sf*-1D, 745 mg; *Sn*-1D, 438 mg; *Sc*-1D, 217 mg; *Sr*-1D, 45.8 mg). Fraction *Sst*-1C was applied to a reverse-phase HPLC column (TSK-gel ODS-120T; 21.5 by 300 mm, Tosoh, Tokyo, Japan) and eluted using a gradient system from CH_3_CN-H_2_O containing 0.2% trifluoroacetic acid (TFA) (1:9) to CH_3_CN-H_2_O containing 0.2% TFA (3:7) to yield the fractions *Sst*-2A to -2Q (134.2 mg), *Sst*-2R (total, 45.1 mg), and *Sst*-2S and -2T (total, 29.4 mg). Similarly, each fraction was applied to a reverse-phase HPLC column (TSKgel ODS-120T, 21.5 by 300 mm; Tosoh, Tokyo, Japan) and eluted using a gradient system from CH_3_CN-H_2_O containing 0.2% TFA (1:4) to CH_3_CN-H_2_O containing 0.2% TFA (3:7) to yield fractions *Sf*-3G (44.7 mg), *Sn*-2E (7.5 mg), *Sn*-3F (23.5 mg), *Sc*-2E (15.2 mg), and *Sc*-3F (24.4 mg).

### Spectroscopic data and properties of *Sst*-2R.

*Sst*-2R is a red-brown powder; ^13^C NMR [acetone-*d*_6_-D_2_O (1:1)]: δ_C_ 167–164 (C-7′′), 158–153 (C-5, 7, 8a), 146–143 (C-3′, 4′, 3′′, 5′′), 140–137 (C-4′′), 132–129 (C-1′), 122–118 (C-6′, 1′′), 117–113 (C-2′, 5′), 111–109 (C-2′′, 6′′), 108–105 (C-8), 98–94 (C-4a, 6), 80–72 (C-2, 3), 35–32 (C-4). Linear TOF-MS: *m/z* 1,455.2, 1,893.6, 2,334.1, 2,773.3, 3,215.1, 3,655.9, 4,094.6; spiral TOF-MS: *m/z* 1,455.1296, 1,895.2106, 2,335.2860, 2,776.3716, 3,217.4494, 3,657.5125, 4,096.5984, 4,537.6521.

### Cells and viruses.

Vero E6/TMPRSS2 cells were purchased from the Japanese Collection of Research Bioresources (Osaka, Japan). SARS-CoV-2 strains (ancestral strain [A lineage]: 2019-nCoV/Japan/TY/WK-521/2020 [GISAID ID: EPI_ISL_408667], Alpha strain [B.1.1.7 lineage]: hCoV-19/Japan/QHN001/2020 [EPI_ISL_804007], Beta strain [B.1.351 lineage]: hCoV-19/Japan/TY8-612-P1/2021 [EPI_ISL_1123289], Gamma strain [P.1 lineage]: hCoV-19/Japan/TY7-501/2021 [EPI_ISL_833366], Delta strain [B.1.617.2 lineage]: hCoV-19/Japan/TY11-927-P1/2021 [EPI_ISL_2158617], and Omicron strain [BA.1 lineage]: hCoV-19/Japan/TY38-873P0/2021 [EPI_ISL_7418017]) were provided by the National Institute of Infectious Diseases (Tokyo, Japan). After inoculation with SARS-CoV-2, Vero E6/TMPRSS2 cells were cultivated in virus growth medium as previously described (39). Dulbecco’s modified Eagle’s medium (Nissui Pharmaceutical Co., Ltd., Tokyo, Japan) supplemented with 1% fetal bovine serum, 2 mM l-glutamine (Fujifilm Wako Pure Chemical Co., Ltd., Osaka, Japan), 100 μg/mL kanamycin (Meiji Seika Pharma Co., Ltd., Tokyo, Japan), and 2 μg/mL amphotericin B (Bristol-Myers Squibb Co., New York, NY) was used as the virus growth medium. HRT-18 Aichi cells and BCoV (Mebus strain) were provided by the Aichi Prefectural Chuo Livestock Hygiene Service Center (Okazaki, Japan). After inoculation with BCoV, HRT-18 Aichi cells were cultivated in virus growth medium with the same composition as that for Vero E6/TMPRSS2 cells, without amphotericin B. MDCK cells were provided by Hideki Nagano (Hokkaido Institute of Public Health, Sapporo, Japan). H1N1 subtype IAV (A/Puerto Rico/8/1934 strain) was purchased from ATCC (Manassas, VA; catalog no. VR-95). After inoculation with IAV, MDCK cells were cultivated in the medium as previously described (40). CRFK cells and FCV (F9 strain) were provided by Ken Maeda (Yamaguchi University, Yamaguchi, Japan). RAW264 cells were obtained from RIKEN BRC (Tsukuba, Japan). MNV (S7 strain) was provided by Yukinobu Tohya (Nihon University, Tokyo, Japan). After inoculation with FCV or MNV, CRFK or RAW264 cells were cultivated in virus growth medium with the same composition as that for Vero E6/TMPRSS2 cells.

### Evaluation of the virucidal activities of sample solutions.

The SARS-CoV-2 ancestral strain, SARS-CoV-2 variant strains, IAV, FCV, and MNV solutions (viral titers, 3.75 to 5.75, 4.75 to 6.25, 3.25 to 5.25, 3.75 to 5.75, and 3.25 to 5.25 log_10_ TCID_50_/mL, respectively) were mixed with *Saxifraga* species-derived rough extracts, *Saxifraga* species-derived fractions, catechin derivatives, DMSO (solvent control for *Saxifraga* species-derived rough extracts, *Saxifraga* species-derived fractions, and catechin derivatives), ethanol, sodium hypochlorite solution, or ultrapure water (solvent control for ethanol and sodium hypochlorite solution). The concentrations of the *Saxifraga* species-derived rough extracts in the mixture were 100 μg/mL. The concentrations of the *Saxifraga* species-derived fractions and catechin derivatives in the mixture were 25 and 100 μg/mL, respectively, when these samples were mixed with SARS-CoV-2, IAV, or FCV and MNV. The concentrations of the ethanol and sodium hypochlorite in the mixture were estimated to be 70% and 2,000 ppm, respectively. The concentration of the sodium hypochlorite solution in the mixture was estimated on the basis of the dilution ratio of the original sodium hypochlorite solution (Fujifilm Wako Pure Chemical Co., Ltd.). The actual free available chlorine concentration was not measured. The mixture of *Saxifraga* species-derived rough extracts and each virus was incubated at 25°C for 1 min. The mixture of *Saxifraga* species-derived fractions, catechin derivatives, ethanol, or sodium hypochlorite solution and SARS-CoV-2, IAV, or FCV was incubated at 25°C for 10 s, while the mixtures of these samples and MNV were incubated at the same temperature for 1 min. To evaluate whether the virucidal activity of *Sst-*2R is concentration and time dependent, the SARS-CoV-2 ancestral strain and MNV solutions were mixed with DMSO or *Sst*-2R at various concentrations for various reaction times. The concentration of *Sst*-2R in the mixture was 25, 50, or 100 μg/mL. The mixture of the test sample and SARS-CoV-2 was incubated at 25°C for 10 s or 15 min, while that of the test sample and MNV was incubated at 25°C for 1 min or 15 min. Following these reactions, the mixtures were inoculated into cells at 10-fold serial dilutions (between 10^1^- and 10^6^-fold dilutions), and the cells were incubated for 3 days at 37°C. In the experiment to evaluate the virucidal activity of sodium hypochlorite solution, the mixtures were inoculated into cells cultured in a medium containing 10 mM sodium thiosulfate to neutralize chlorine. The viral titer in each group was evaluated as previously described (11). The reduction in viral titer by each sample treatment was calculated as follows: (viral titer in the solvent control group) − (viral titer in each sample group).

### Sample solution cytotoxicity evaluation.

The *Sst*-1C, *Sst*-1D, and *Sst*-2R solutions were 2-fold serially diluted (from 100.0 to 0.1 μg/mL) with phosphate-buffered saline (PBS). The ethanol and sodium hypochlorite solutions were also 2-fold serially diluted (from 70.0% to 0.3% and from 2,000 ppm to 7.8 ppm, respectively) with PBS. Vero E6/TMPRSS2, MDCK, CRFK, and RAW264 cells were incubated in these diluted solutions or PBS without test sample at 37°C for 1 h. After the incubation, the solutions were replaced with a test sample-free cell culture medium. After 3 days of incubation at 37°C, the cell viability was evaluated using the CellTiter-Glo luminescent cell viability assay (Promega Co., Madison, WI), and the CC_50_ of each test sample was calculated using nonlinear regression analysis. The difference between the concentration showing virucidal activity in the virucidal test (Table 1 and Table S3) and the CC_50_ of each test sample was evaluated using the following formula: concentration showing virucidal activity in virucidal test/CC_50_ in cytotoxicity test. The concentration of each sample that showed virucidal activity in the virucidal test was as follows: *Sst*-1C, *Sst*-1D, and *Sst*-2R, 25 μg/mL (target viruses: SARS-CoV-2, IAV, and FCV) and 100 μg/mL (MNV); ethanol, 70% (SARS-CoV-2, IAV, FCV, and MNV); and sodium hypochlorite solution, 2,000 ppm (SARS-CoV-2, IAV, FCV, and MNV). As shown in Table S5, the concentration that showed virucidal activity in the virucidal test against SARS-CoV-2, IAV, FCV, and MNV was compared with the CC_50_ in the cytotoxicity test against Vero E6/TMPRSS2, MDCK, CRFK, and RAW264 cells, respectively. An example of the calculation using the above formula is as follows (in the case of *Sst*-1C, with SARS-CoV-2 as the target virus in the virucidal test and with Vero E6/TMPRSS2 cells as the target cell in the cytotoxicity test): (concentration showing virucidal activity in virucidal test)/(CC_50_ in cytotoxicity test) = (25 μg/mL)/(20.7 μg/mL) = 1.2 (Table S5).

### Evaluation of the virucidal activities of sample creams.

Solutions of the SARS-CoV-2 ancestral strain, IAV, FCV, and MNV (viral titers, 3.75 to 5.25, 3.75 to 6.25, 4.25 to 5.25, and 3.75 to 5.25 log_10_ TCID_50_/mL, respectively) were treated with base cream (control) or 5% or 10% *Sst*-1D-containing cream. The base of the cream was prepared by mixing glycerol (Fujifilm Wako Pure Chemical Co., Ltd.) and white petroleum jelly (Fujifilm Wako Pure Chemical Co., Ltd.) in a 1:9 weight ratio. A total of 10 mg of each cream was applied to 2.25 cm^2^ (1.5 cm by 1.5 cm) of polyethylene terephthalate film (AS ONE Co., Ltd., Osaka, Japan). Sixty microliters of virus solution was covered by this film and incubated at 25°C for 10 min. The virus solution was then collected, and the viral titer in each group was evaluated as previously described (34). The reduction in viral titer produced by each sample cream treatment was calculated as follows: (viral titer in base cream group) − (viral titer in each sample cream group).

### Effect of *Saxifraga* species-derived condensed tannins on viral structural proteins.

The SARS-CoV-2 ancestral strain or IAV (viral titer, 6.75 or 5.75 log_10_ TCID_50_/mL, respectively) was mixed with DMSO (solvent control) or 25 μg/mL *Sst*-2R. MNV (viral titer, 6.25 log_10_ TCID_50_/mL) was mixed with DMSO or 100 μg/mL *Sst*-2R. The mixture containing SARS-CoV-2 or IAV was incubated at 25°C for 10 s, and that containing MNV was incubated for 1 min. After each reaction time, SDS buffer with 2-mercaptoethanol (Fujifilm Wako Pure Chemical Co., Ltd.) was added to the mixtures. The mixtures were heated at 100°C for 2 min and then subjected to SDS–PAGE. The protein bands in the gel were transferred to a polyvinylidene difluoride membrane (Bio-Rad Laboratories Inc., Hercules, CA). The membrane was blocked using 2% skim milk–PBS with 0.05% polyoxyethylene sorbitan monolaurate (Fujifilm Wako Pure Chemical Co., Ltd.). After blocking, each membrane was reacted with each of the primary antibodies as follows. SARS-CoV-2/2019-nCoV spike antibody, rabbit polyclonal antibody (Sino Biological Inc., Beijing, China), IAV H1N1 (A/Puerto Rico/8/1934) HA antibody, rabbit polyclonal antibody (antigen affinity purified; Sino Biological Inc.), and mouse anti-MNV-1 monoclonal antibody (Merck & Co., Inc., Kenilworth, NJ) were used as primary antibodies for the detection of SARS-CoV-2 S protein S1 subunit, IAV HA, and MNV VP1, respectively. After the reaction, each membrane was reacted with each of the secondary antibodies as follows. Mouse anti-rabbit IgG peroxidase conjugate (Sino Biological Inc.) and horseradish peroxidase-conjugated goat anti-mouse IgG2b (Thermo Fisher Scientific Inc., Waltham, MA) were used for the detection of SARS-CoV-2 S protein S1 subunit and IAV HA and MNV VP1, respectively (11, 34). After the reaction, the membranes were reacted with enhanced chemiluminescence (ECL) Prime WB detection reagent (GE Healthcare Ltd., Chicago, IL), and the chemiluminescence was detected using the LAS-3000 imaging system (Fujifilm Co., Ltd., Tokyo, Japan).

In another experiment, DMSO- or *Sst*-2R-treated BSA was detected using Western blot analysis. BSA dissolved in ultrapure water was mixed with DMSO or *Sst*-2R. The concentration of BSA in the mixture was 6 μg/mL, and that of *Sst*-2R was 25 μg/mL or 100 μg/mL. After 10 s, SDS-PAGE and Western blotting targeting BSA were performed as explained above. BSA polyclonal antibody (Bioss Antibodies, Inc., Woburn, MA) and mouse anti-rabbit IgG peroxidase conjugate were used as the primary and secondary antibodies, respectively.

### Analysis of the morphology of viral particles treated with *Saxifraga* species-derived condensed tannins.

BCoV or IAV (viral titer, 4.75 or 5.75 log_10_ TCID_50_/mL, respectively) was mixed with DMSO (solvent control) or 20 μg/mL or 100 μg/mL *Sst*-2R. After 10 s or 3 h at 25°C, negatively stained samples were prepared for TEM, and the viral particles were observed using TEM as previously described (41). Because SARS-CoV-2 could not be used in this analysis owing to safety considerations in handling pathogens in our facility, BCoV, which belongs to the genus *Betacoronavirus* like SARS-CoV-2, was used as the surrogate virus. MNV (viral titer, 6.25 log_10_ TCID_50_/mL) was mixed with DMSO or 100 μg/mL *Sst*-2R. After 1 min or 3 h at 25°C, the viral particles were observed using TEM, as explained above. Through the TEM observation of negatively stained viral particles, particles of normal size and those that did not have obviously abnormal spike proteins, capsids, or envelopes were considered intact.

### Statistical analysis.

To evaluate the virucidal activity of the sample solutions and creams, Student’s *t* tests were used to analyze the statistical significance of the differences in the viral titers between the control and each sample group; a *P* value of <0.05 was considered statistically significant.
