# Supplementary material for: Rapid Virucidal Activity of Japanese Saxifraga Species-Derived Condensed Tannins against SARS-CoV-2, Influenza A Virus, and Human Norovirus Surrogate Viruses
Source: Appl Environ Microbiol. 2023 May 15;89(6):e00237-23. doi: 10.1128/aem.00237-23 (PMC10304655; doi:10.1128/aem.00237-23)
Supplement: Supplemental file 1 — Supplemental material. Download aem.00237-23-s0001.pdf, PDF file, 1.3 MB [file aem.00237-23-s0001.pdf]

Supplemental material for **Rapid virucidal activity of Japanese *Saxifraga* species-derived condensed tannins against SARS-CoV-2, influenza A virus, and human norovirus surrogate viruses**

Toshihiro Murata,[a] Dulamjav Jamsransuren,[b] Sachiko Matsuda,[b] Haruko Ogawa,[b] Yohei Takeda[b,c]

[a]Division of Pharmacognosy, Tohoku Medical and Pharmaceutical University, Sendai, Miyagi, Japan; murata-t@tohoku-mpu.ac.jp

[b]Department of Veterinary Medicine, Obihiro University of Agriculture and Veterinary Medicine, Obihiro, Hokkaido, Japan; jduuya@obihiro.ac.jp (D.J.); chaka@obihiro.ac.jp (S.M.); hogawa@obihiro.ac.jp (H.O.); ytakeda@obihiro.ac.jp (Y.T.)

[c]Research Center for Global Agromedicine, Obihiro University of Agriculture and Veterinary Medicine, Obihiro, Hokkaido, Japan

Address correspondence to Yohei Takeda: ytakeda@obihiro.ac.jp

Toshihiro Murata and Yohei Takeda contributed equally to this work. Author order was determined in order of increasing seniority.

Table S1. Virucidal activities of *Saxifraga* species-derived rough extracts against multiple virus species

	SARS-CoV-2[a] (ancestral strain)	IAV	FCV	MNV
	100 μg/ml[b]	100 μg/ml	100 μg/ml	100 μg/ml
	1 min[c]	1 min	1 min	1 min
Sst-rough extract[d]	≥2.75 ± 0.65[e]**	≥1.25 ± 0.29**	2.50 ± 0.58**	–0.38 ± 0.48[ns]
Sf-rough extract	≥3.38 ± 0.63**	≥1.50 ± 0.29**	2.00 ± 0.41**	–0.13 ± 0.85[ns]
Sr-rough extract	≥3.38 ± 0.63**	≥2.13 ± 0.25***	≥2.75 ± 0.50**	1.00 ± 0.41*
Sn-rough extract	0.38 ± 0.65[ns]	0.13 ± 0.25[ns]	0.75 ± 0.54**	–0.25 ± 0.65[ns]
Sc-rough extract	≥3.00 ± 0.71**	≥1.25 ± 0.29**	2.38 ± 0.48**	–0.75 ± 0.65[ns]

a) Target virus, b) concentration of samples, c) reaction time, d) sample name, e) reduction in viral titer ($\log_{10}$ $TCID_{50}$/ml): [viral titer of DMSO group] – [viral titer of sample group] ± S.D. The degree of the reduction in the viral titer represents the degree of virucidal activity of each test sample. Statistical analysis (Student's *t*-test; n = 4–10): *$p < 0.05$, **$p < 0.01$, ***$p < 0.001$, ns: not significant. Light gray and dark gray highlights indicate statistically significant difference and no significant differences, respectively.
Sst: *S. stolontifera*; *Sf*: *S. fortunei*; *Sr*: *S. rebunshirensis*; *Sn*: *S. nipponica*; *Sc*: *S. cortusifolia*

Table S2. Virucidal activities of *Sst*-2R at various concentrations against SARS-CoV-2 for various reaction times

		SARS-CoV-2[a] (ancestral strain)		MNV	
		10 s[b]	15 min	1 min	15 min
Sst-2R[c]	25 μg/ml[d]	≥2.88 ± 0.48[e]**	≥3.75 ± 0.65**	0.88 ± 0.48*	1.50 ± 0.00***
	50 μg/ml	≥3.13 ± 0.25**	≥4.38 ± 0.63***	1.38 ± 0.48*	≥2.13 ± 0.25***
	100 μg/ml	≥3.75 ± 0.29***	≥4.50 ± 0.58***	1.50 ± 0.58*	≥2.25 ± 0.29***

a) Target virus, b) reaction time, c) sample name, d) concentration of the sample, e) reduction in viral titer ($\log_{10}$ $TCID_{50}$/ml): [viral titer of DMSO group] − [viral titer of sample group] ± S.D. The degree of the reduction in the viral titer represents the degree

of virucidal activity of each test sample. Statistical analysis (Student's *t*-test; $n = 4$): *$p < 0.05$, **$p < 0.01$, ***$p < 0.001$. Light gray highlights indicate statistically significant differences. *Sst*: *S. stolontifera*

Table S3. Virucidal activities of 70% ethanol and 2,000 ppm sodium hypochlorite solution against multiple virus species

	SARS-CoV-2[a] (ancestral strain)	IAV	FCV	MNV
	10 s[b]	10 s	10 s	1 min
70% EtOH[c]	≥4.17 ± 0.26[d]***	≥4.00 ± 0.00***	2.86 ± 0.24***	≥3.00 ± 0.00***
2,000 ppm NaClO	≥4.17 ± 0.26***	≥4.00 ± 0.00***	≥3.93 ± 0.19***	≥3.13 ± 0.25***

a) Target virus, b) reaction time, c) sample name, d) reduction in viral titer ($\log_{10}$ $TCID_{50}$/ml): [viral titer of ultrapure water group] – [viral titer of sample group] ± S.D. The degree of the reduction in the viral titer represents the degree of virucidal activity of each test sample. Statistical analysis (Student's *t*-test; $n = 4–7$): ***$p < 0.001$. Light gray highlights indicate statistically significant differences.
EtOH: ethanol; NaClO: sodium hypochlorite solution

Table S4. Virucidal activities of catechin derivatives against multiple virus species

	SARS-CoV-2[a] (ancestral strain)	IAV	FCV	MNV
	25 μg/ml[b]	25 μg/ml	25 μg/ml	100 μg/ml
	10 s[c]	10 s	10 s	1 min
Catechin[d]	–0.25 ± 0.29[e]ns	0.25 ± 0.65ns	–0.13 ± 0.75ns	0.25 ± 0.50ns
Epicatechin	0.00 ± 0.41ns	0.00 ± 1.00ns	0.25 ± 0.96ns	0.13 ± 0.48ns
Epigallocatechin	0.00 ± 0.00ns	0.25 ± 0.87ns	–0.25 ± 0.87ns	0.50 ± 0.41ns
Epicatechin gallate	–0.13 ± 0.75ns	0.00 ± 1.00ns	–0.13 ± 0.75ns	0.50 ± 0.71ns
Epigallocatechin gallate	0.63 ± 0.75ns	0.13 ± 0.48ns	0.13 ± 0.85ns	0.13 ± 0.25ns

a) Target virus, b) concentration of samples, c) reaction time, d) sample name, e) reduction in viral titer ($\log_{10}$ $TCID_{50}$/ml): [viral titer of DMSO group] – [viral titer of sample group] ± S.D. The degree of the reduction in the viral titer represents the degree of virucidal activity of each test sample. Statistical analysis (Student's *t*-test; $n = 4$): ns: not significant.

Dark gray highlights indicate no statistically significant differences.

Table S5. Cytotoxicity of *Saxifraga* species-derived fractions, ethanol, and sodium hypochlorite solution

	Target cell: Vero E6/TMPRSS2 cell		
	a: Concentration showing virucidal activity in virucidal test (Table 1 and Supplementary Table 3)	b: CC_{50} in the cytotoxicity test	a/b
Sst-1C	25 μg/ml	20.7 μg/ml	1.2
Sst-1D	25 μg/ml	21.9 μg/ml	1.1
Sst-2R	25 μg/ml	13.7 μg/ml	1.8
EtOH	70%	6.7%	10.5
NaClO	2,000 ppm	226.3 ppm	8.8

	Target cell: MDCK cell		
	a: Concentration showing virucidal activity in virucidal test (Table 1 and Supplementary Table 3)	b: CC_{50} in the cytotoxicity test	a/b
Sst-1C	25 μg/ml	19.5 μg/ml	1.3
Sst-1D	25 μg/ml	21.9 μg/ml	1.1
Sst-2R	25 μg/ml	15.6 μg/ml	1.6
EtOH	70%	4.5%	15.6
NaClO	2,000 ppm	200.2 ppm	10.0

	Target cell: CRFK cell		
	a: Concentration showing	b: CC_{50}	a/b

	virucidal activity<br>in virucidal test<br>(Table 1 and<br>Supplementary Table 3)	in the cytotoxicity<br>test	
Sst-1C	25 µg/ml	22.2 µg/ml	1.1
Sst-1D	25 µg/ml	22.7 µg/ml	1.1
Sst-2R	25 µg/ml	13.9 µg/ml	1.8
EtOH	70%	5.4%	13.0
NaClO	2,000 ppm	131.2 ppm	15.2

	Target cell: RAW264 cell		
	a: Concentration showing<br>virucidal activity<br>in virucidal test<br>(Table 1 and<br>Supplementary Table 3)	b: CC_{50}<br>in the cytotoxicity<br>test	a/b
Sst-1C	100 µg/ml	8.3 µg/ml	12.1
Sst-1D	100 µg/ml	9.9 µg/ml	10.1
Sst-2R	100 µg/ml	9.3 µg/ml	10.8
EtOH	70%	6.2%	11.3
NaClO	2,000 ppm	143.3 ppm	14.0

Sst: *S. stolontifera*: EtOH: ethanol; NaClO: sodium hypochlorite solution

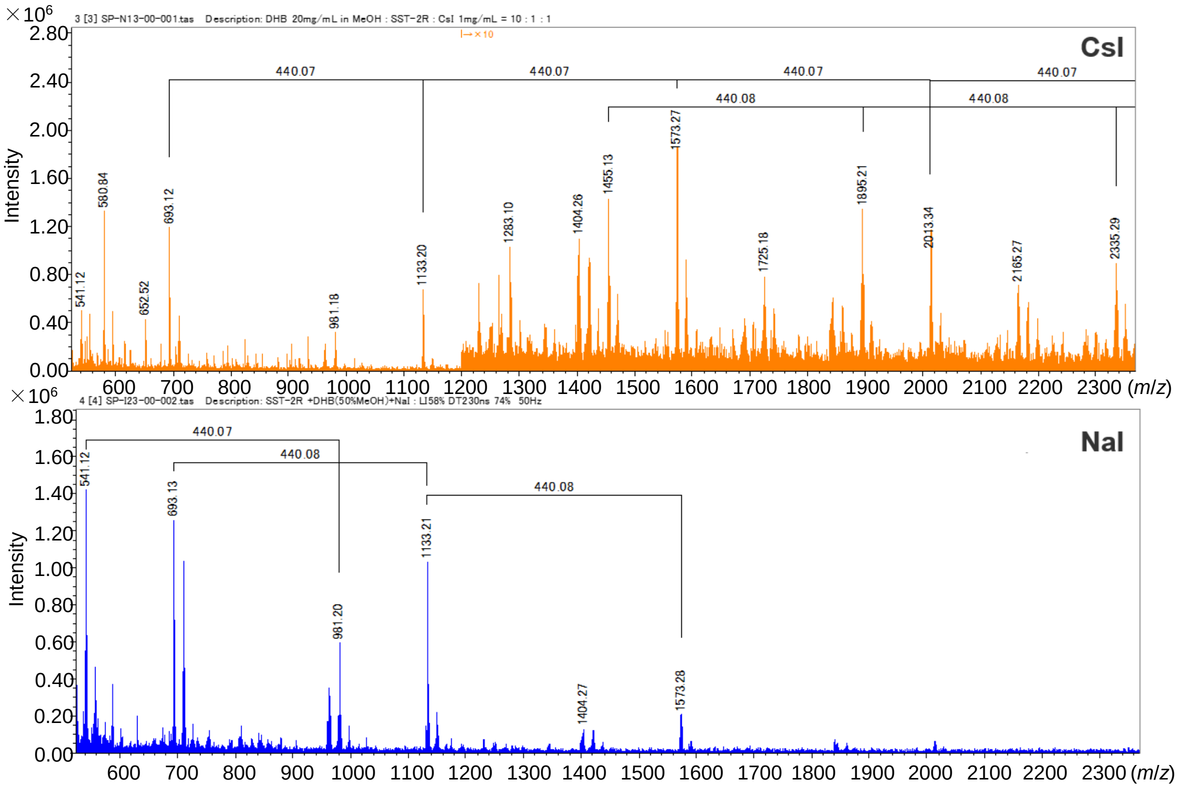

Fig. S1. Spiral mode TOF-MS spectra of *Sst*-2R using cesium iodide or sodium iodide as cationization agents.

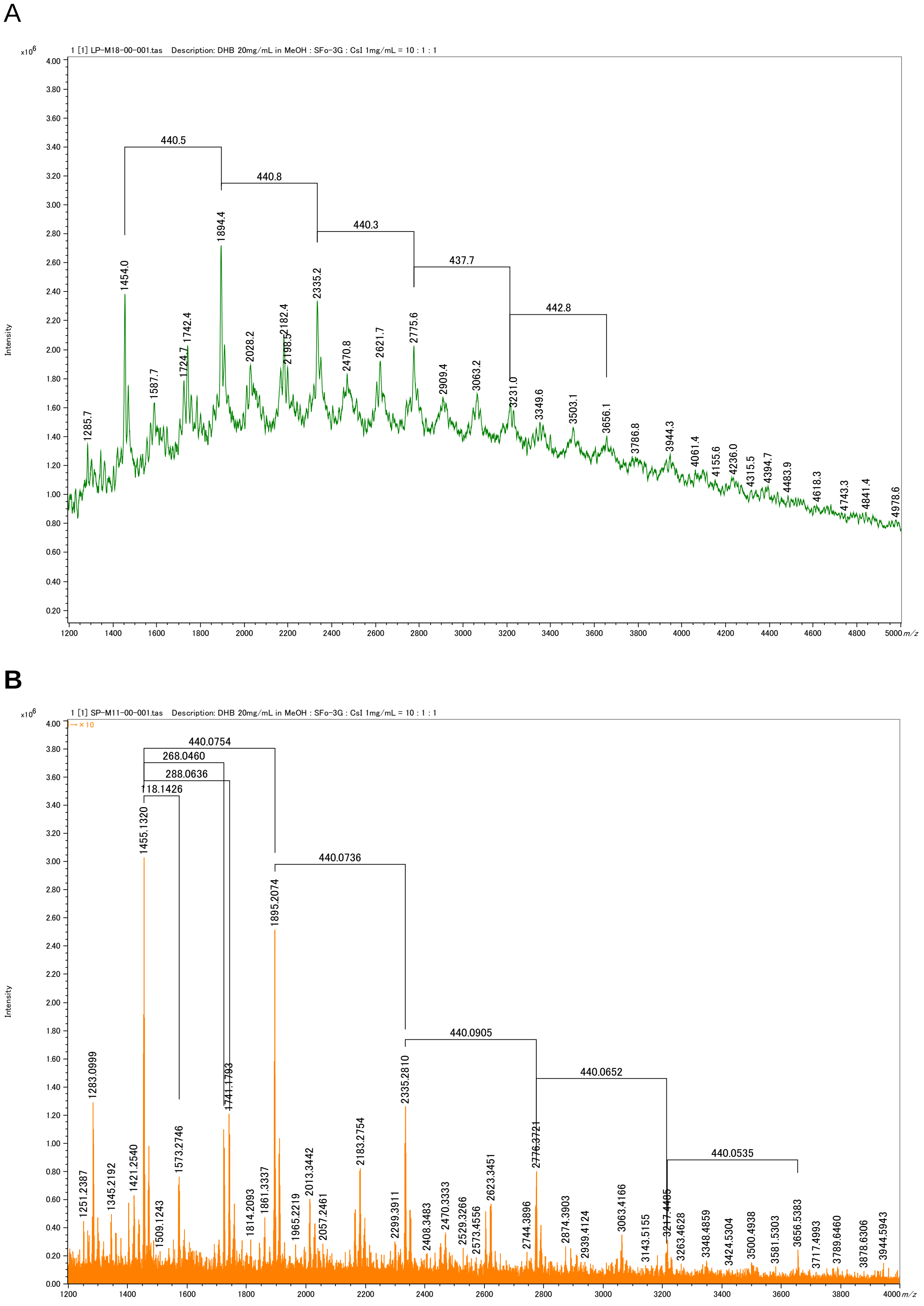

Fig. S2. Linear (A) and spiral (B) mode TOF-MS spectra of *Sf*-3G.

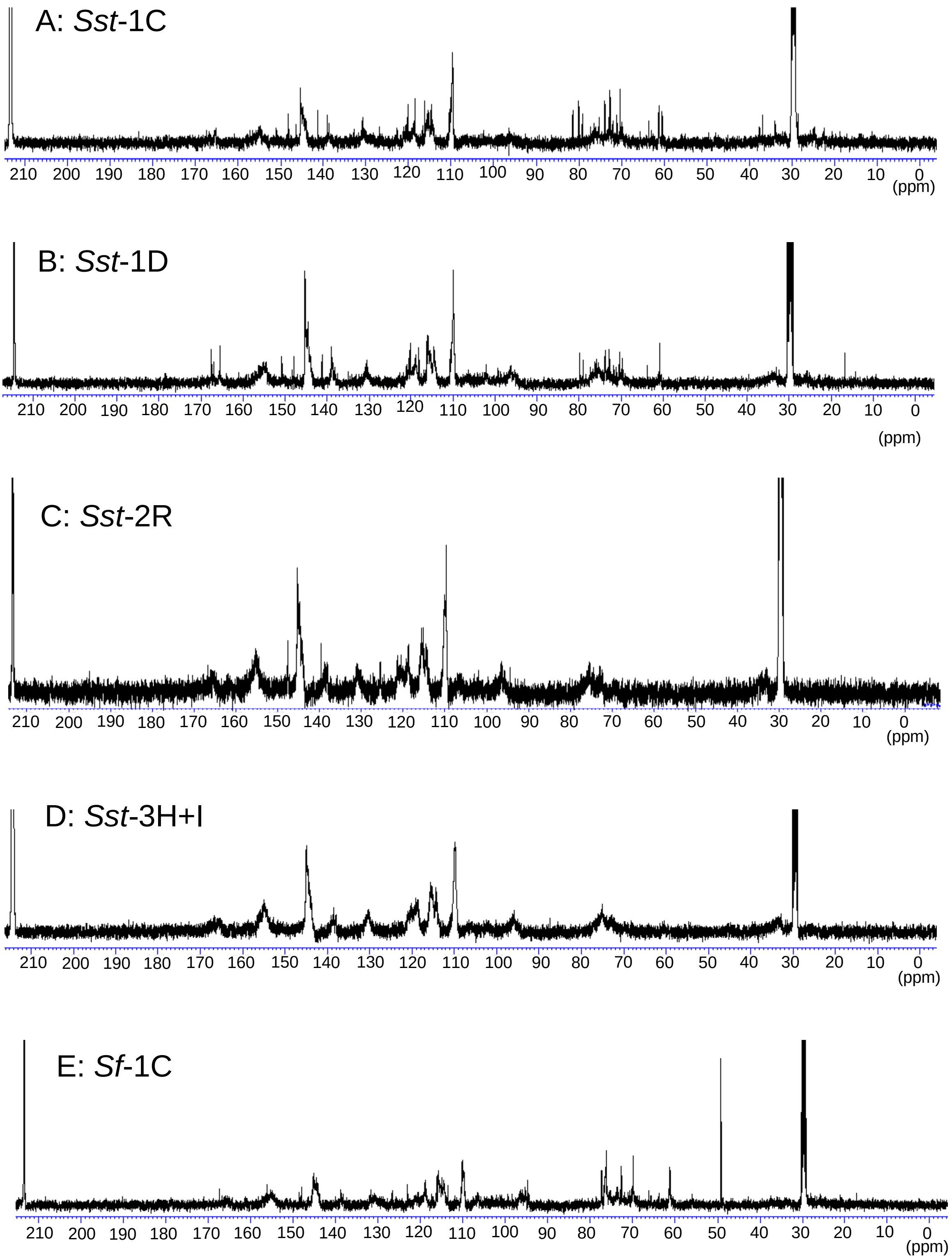

Fig. S3. The ^{13}C NMR spectra [acetone-d_6-D_2O (1:1), 100MHz] of *Sst*-1C (A), *Sst*-1D (B), *Sst*-2R (C), *Sst*-3H+I (D), *Sf*-1C (E), *Sf*-1D (F), *Sf*-2H (G), *Sf*-3G (H), *Sn*-1C (I), *Sn*-1D (J), *Sn*-2E+3F (K), *Sc*-1C (L), *Sc*-1D (M), *Sc*-2E+3F (N), *Sr*-1C (O), and *Sr*-2E (P).

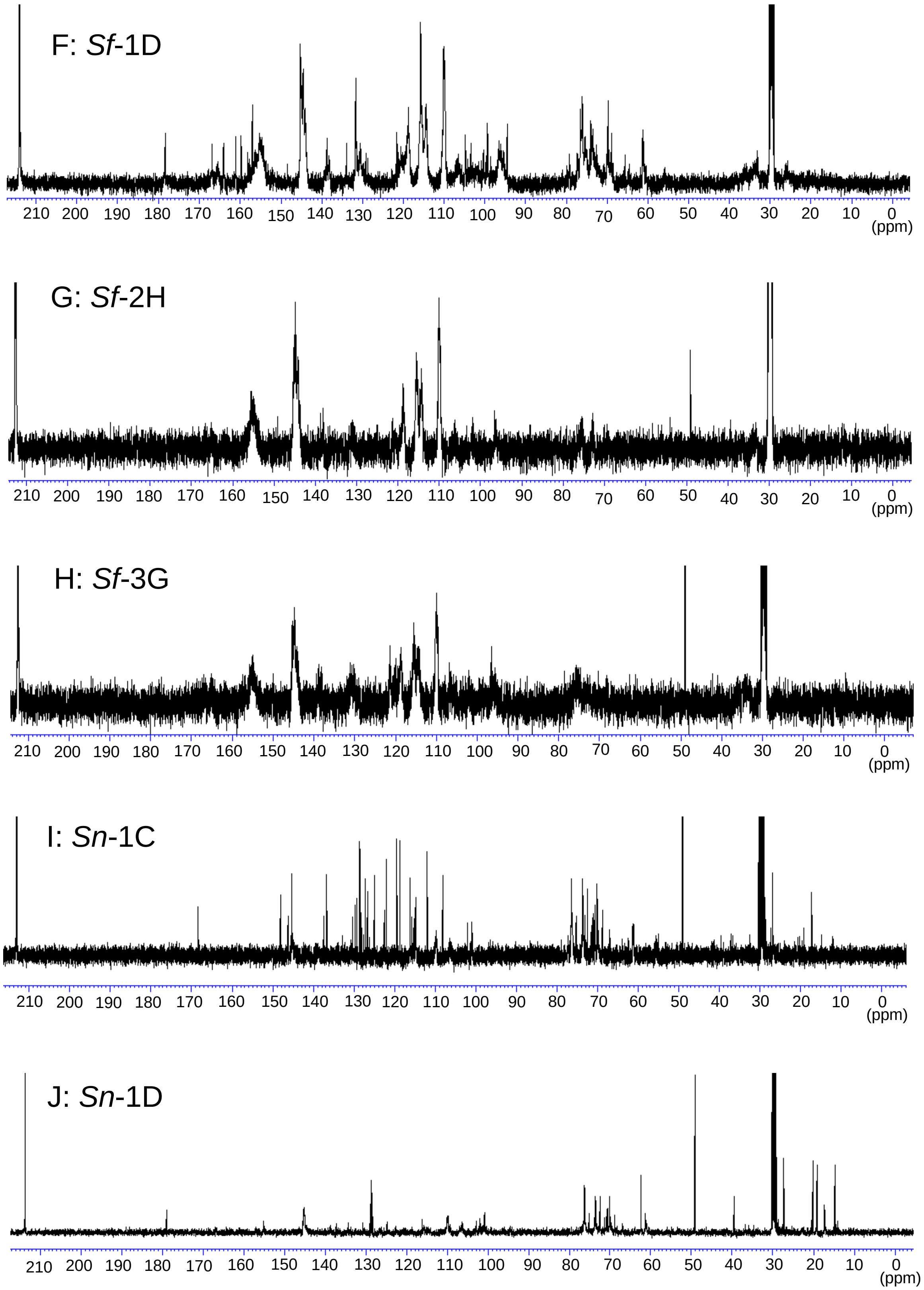

Fig. S3. Continued.

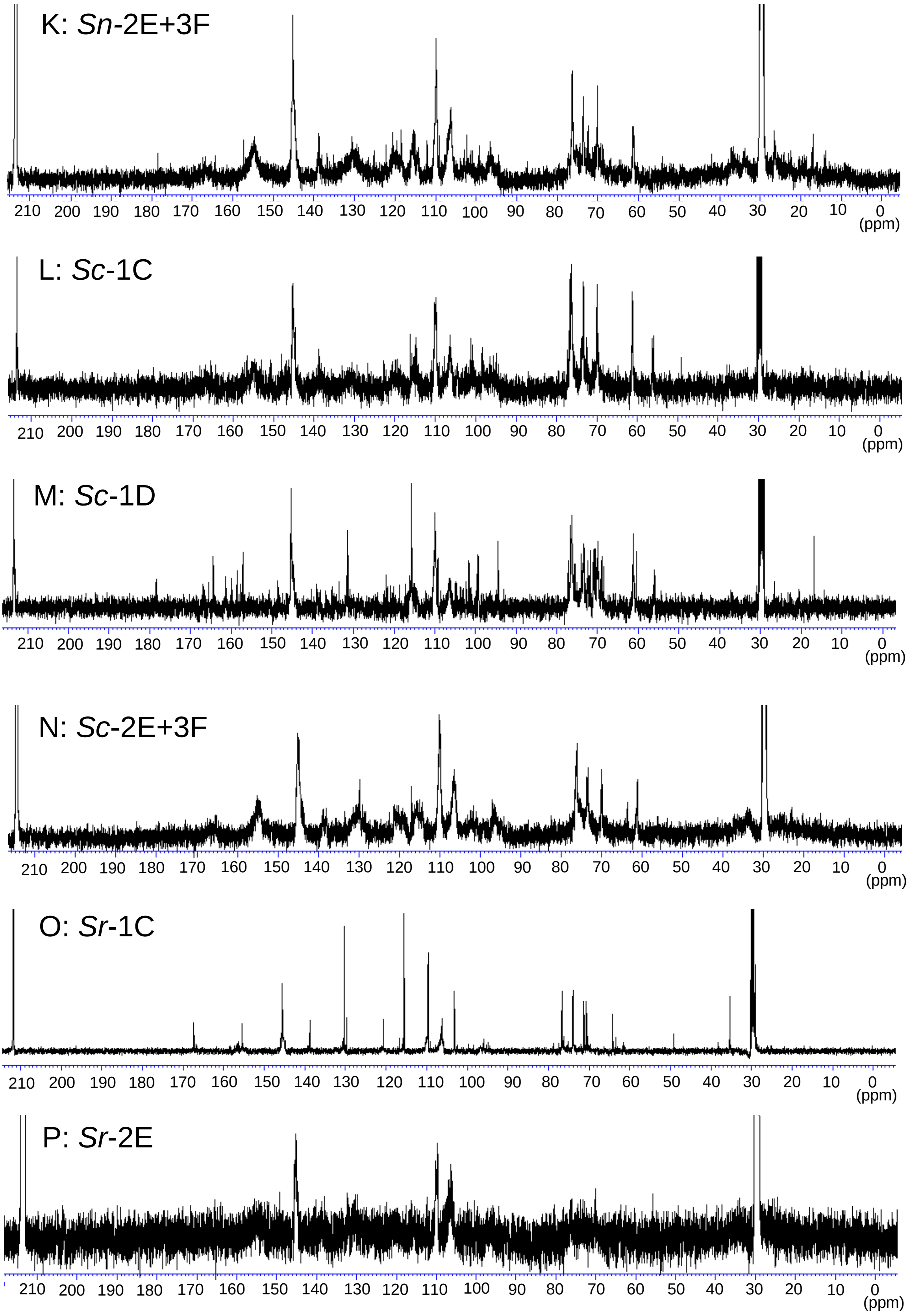

Fig. S3. Continued.

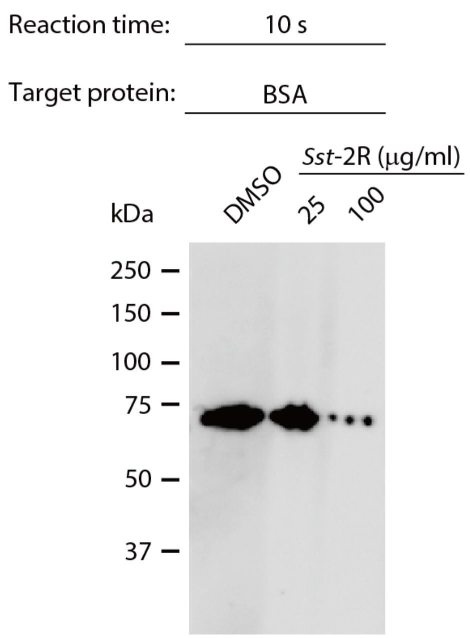

Fig. S4. Effect of *Sst*-2R on BSA. BSA solution was mixed with DMSO or *Sst*-2R. The concentration of BSA in the mixture was 6 µg/ml and that of *Sst*-2R was 25 µg/ml or 100 µg/ml. After 10 s, Western blotting targeting BSA was visualized.

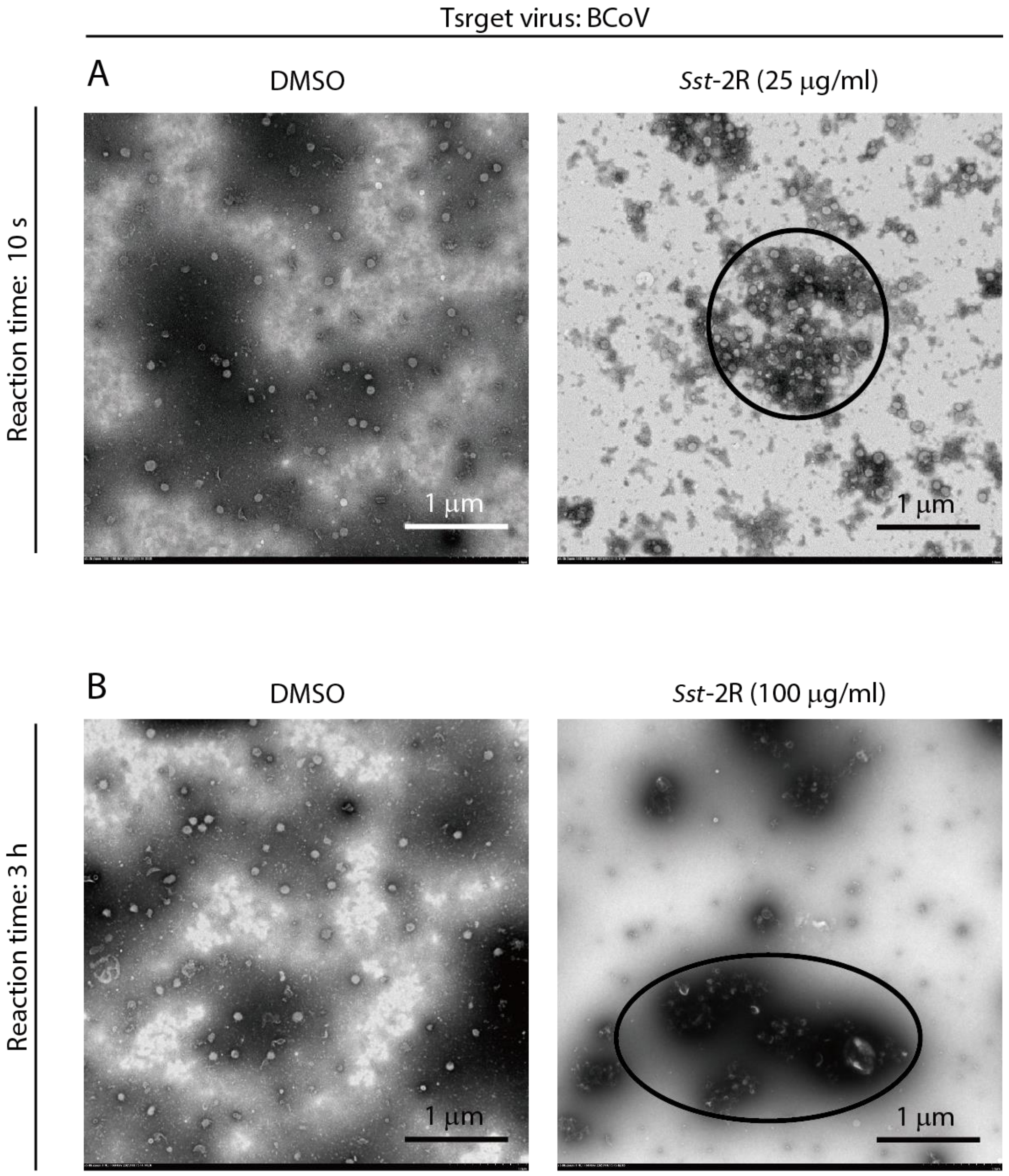

Fig. S5. Morphology of *Sst*-2R-treated BCoV particles (low-power field of view). (A, B) BCoV solution was mixed with DMSO or *Sst*-2R. The concentration of *Sst*-2R in the mixture was 25 μg/ml (A) or 100 μg/ml (B). After 10 s (A) or 3 h (B), the viral particles were observed using TEM. TEM images with low-power field of view (35 μm^2) are shown. Black round frame: aggregated viral particles.

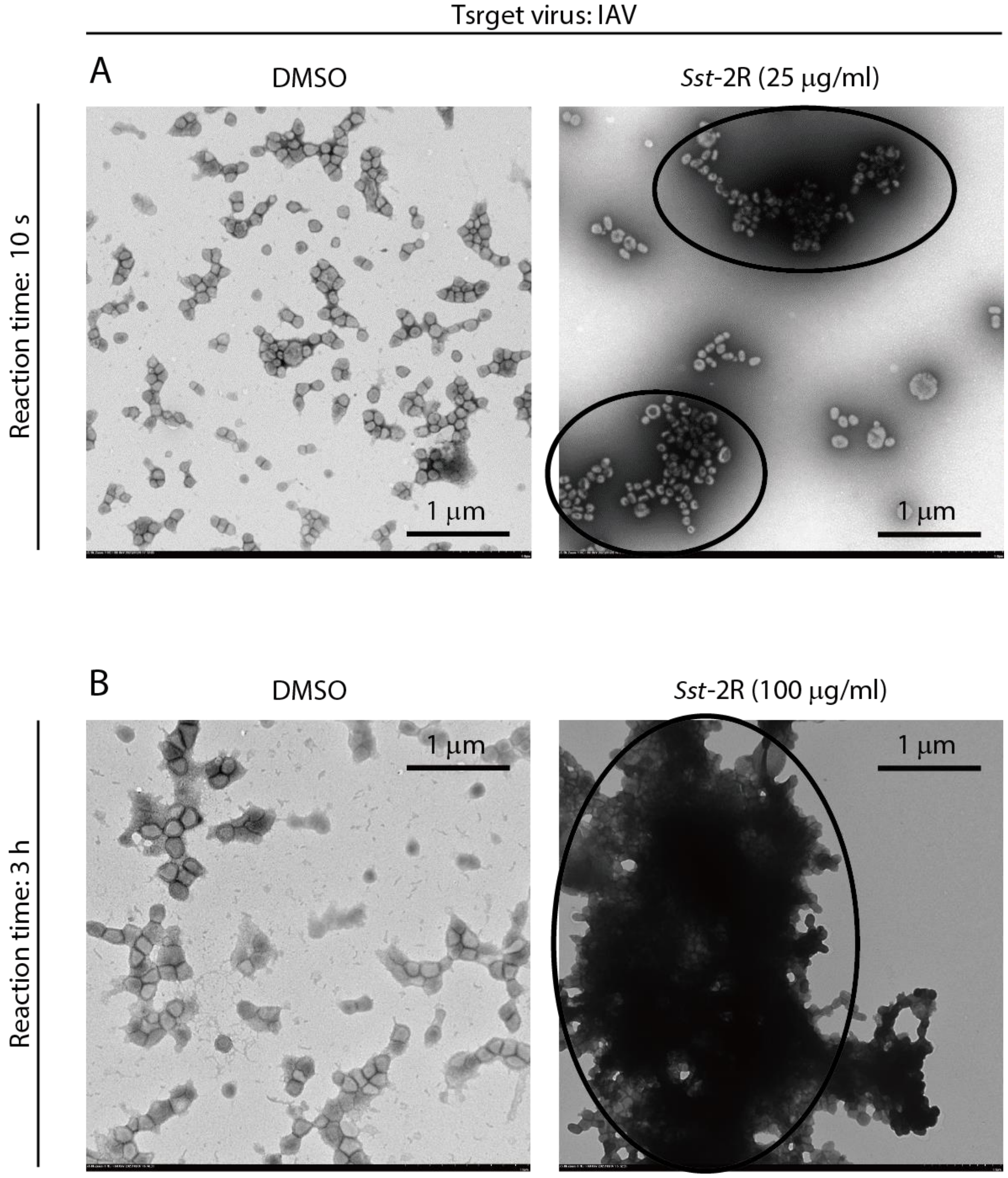

Fig. S6. Morphology of *Sst*-2R-treated IAV particles (low-power field of view). (A, B) IAV solution was mixed with DMSO or *Sst*-2R. The concentration of *Sst*-2R in the mixture was 25 μg/ml (A) or 100 μg/ml (B). After 10 s (A) or 3 h (B), the viral particles were observed using TEM. TEM images with low-power field of view (35 μm^2) are shown. Black round frame: aggregated viral particles.

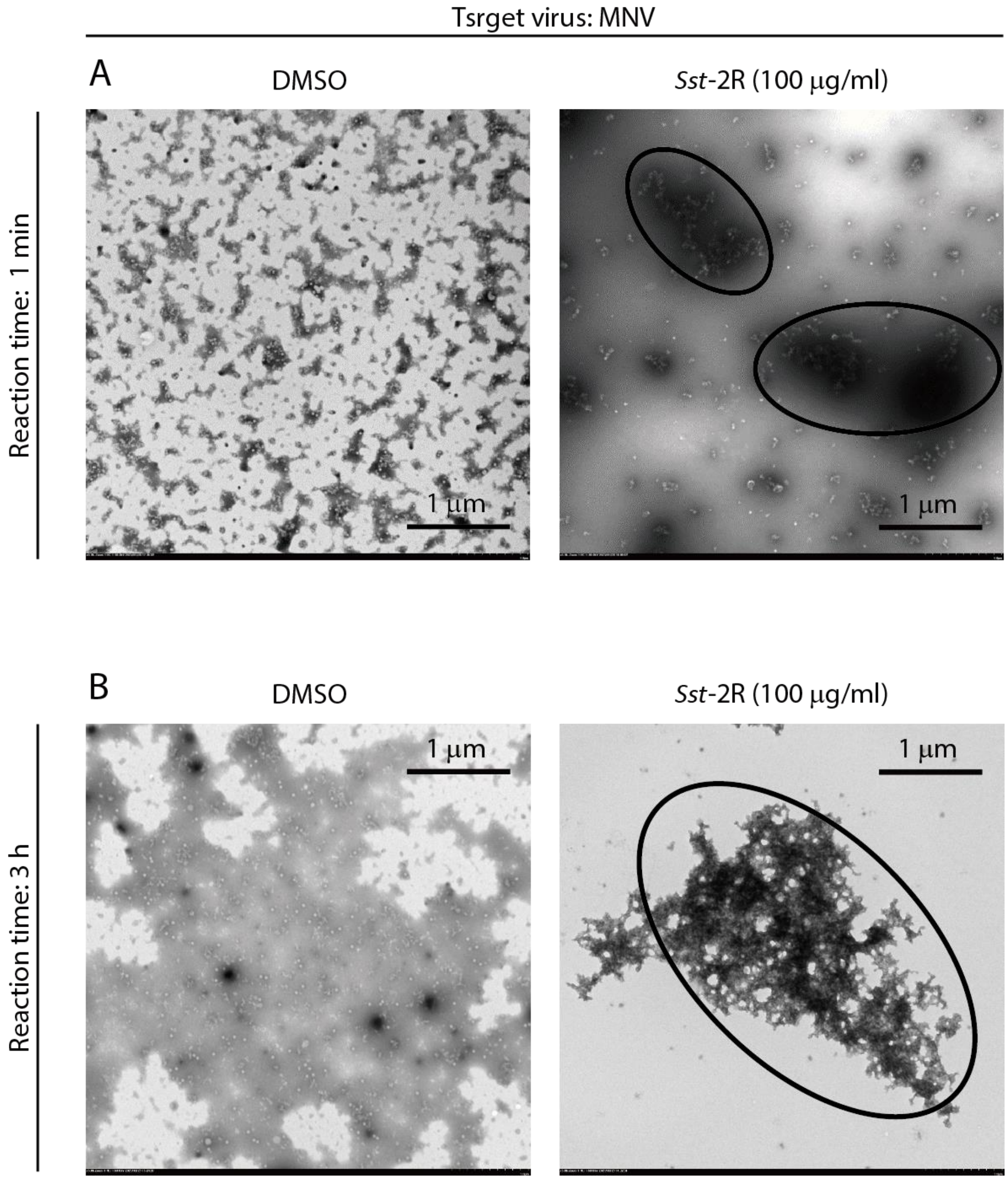

Fig. S7. Morphology of *Sst*-2R-treated MNV particles (low-power field of view). (A, B) MNV solution was mixed with DMSO or *Sst*-2R. The concentration of *Sst*-2R in the mixture was 100 µg/ml. After 1 min (A) or 3 h (B), the viral particles were observed using TEM. TEM images with low-power field of view (35 μm^2) are shown. Black round frame: aggregated viral particles.

The nucleotide sequences of internal transcribed spacer 1.

Saxifraga stolonifera

GGCGAAAATCCGTTGCCGAGAGTCATTAAAGACATCAAGAAGATGACATCCT
TCCGAAATGTTGCACGAGCAAACCACGGAACAGATGCACATTCTTTTAATTT
CCTTGGCGCATTTCACGCCGGGTTTTTGTTTCATGTCAAGTCAACCCGGGAA
AACGCAGCTCAAACGACGGGCGTTCGGGTGCTGCGTGTTCACCGGGCAGGC
CTCGACATTGGGAACGAAGGGCGGCATGCCAAAGAAGACAGCGCCTTTAGT
TCGTAGGGTTGCGATTACGTGTTCACAAGTTGTTCTGCTTTGAACCAGGGG

Saxifraga fortunei

GATGCGAGAGCCGAGATATCCGTTGCCGAGAGTCATTATAGACATCAAGAAG
ATAACATCGTTCCGAAATATTATGCAAGCAAAACACGGGACGGATGCACACT
CTTTTACATTCCTTGGCGCATTTCACGCCGGGTTTTTGTTTTATATCAAGTCAA
GCCGGGCAACCGCAACTCAAACGGCAAGCGTTAATGGGCTGCGATATGACC
AAGCATGCCTTGACATTGAGAACAGAGGTTGCCCGCCAAATGCGACGGCCC
CTTAGTTCATCGGTTGTGATTACATGTTCACAAGTTGTTCTGCTTTGAGCAAG
TGGCGACAATGATCCTTCCGCAGGTTCACCTAC

Saxifraga nipponica

GATGCGAGAGCCGAGATATCCGTTGCCGAGAGTCATTAAAGACATCAAGAAG
ATGACATCCTTCCGAAATGTTGCGTAAGCAAACACAGGATGGATGCACGTTC
TTTTAAGTTCCTTGGCGCATTTCACGCCGGGTTTTTGTTGTATCAAGTCAACC
CGGAACAGGCAGCTCAAACGACGGGCATCGATGGGCTACGTTATTACCGAGC
AGGCCTCGACATTGGGAACAAAGGGCGACCTGCCAAATGAGACTGGGCCCT
TAGTTCCTAGGTTGCGATTACATGTTCACAAGTTGTTCTGCTTTGAGCAGGTG
GCGACAATGATCCTTCCGCAGGTTCACCTACGGAAACCTTGTTAC

Saxifraga cortusifolia

TCGTTCTTCATCGATGCGAGAGCCGAGATATCCGTTGCCGAGAGTCATTAAAG
ACATCAAGAAGATGACATCCTTCCGAAATGTTGCATAAGCAAACACAGGATG
GATGCACATTCTTTTAAGTTCCTTGGCGCATTTCACGCCGGTTTTTTGTTTTAT
CAAGTCAACCCGGAAAACGCAGCTCAAACGACTGGCATCGATGGGCTACGT
TATTACCGAGAGGCCTTGACATTGGGAACAAAGGGCAACCTGCCAAATGAG
ACATGGCCCTTAGTTCGTAAGTTGCGATTACATGTTCACAAGTTGTTCTGCCT
TGAGCAGGTGGCGACAATGATCCTTCCGCAGGTTCACCTACGGAAACCTTGT
TACGAG

Saxifraga rebunshirensis

CCGAAAGTCCATTGAACCTTATCATTTAGAGGAAGGAGAAGTCGTAACAAGG
TTTCCGTAGGTGAACCTGCGGAAGGATCATTGTCGTAACCTGCTAAGGCAAA
ACAACCTGCGAACATGTACTTACATTCGGGGATCCAAGTGCGTCTTCTTCGGC
GGATGCTCTTTCTTCCCAACGTTGAGATGGCTCGGTGTGTGTTTTGCTTACCA
ACGAAGTATTCGTTGAGAGTTGGCACTCACTGAGCTTTCTCGACATAACAAA
AACCCGGCGTGAACTGCGCCAAGGAACATAATAAGATCGTGCATTCTCCCGG
TGTTTGCTTTTGCTTCACTCGGACGGATGTCATCTTCTTGATGAGTTTATAATG
ACTCTCGGCAACGGATATCTCGGCTCTCGCATCATTGAAAGAACGAGA

Extraction and fractionation procedures

Dried *S. stolonifera* (10 g) was extracted using acetone-H_2O (4:1) at room temperature for 4 weeks. After concentration, 2.6 g of the rough extract was obtained. The extract was dissolved in H_2O, and the solution was applied to a porous polymer gel (Diaion HP-20, 60 g) open column, and eluted with H_2O (fraction *Sst*-1A, 1.55 g), methanol (MeOH)-H_2O (1:4) (fraction *Sst*-1B, 101 mg), MeOH-H_2O (2:3) (fraction *Sst*-1C, 282 mg), MeOH-H_2O (3:2) (fraction *Sst*-1D, 355 mg), MeOH-H_2O (9:1) (fraction *Sst*-1E, 35.2 mg), and MeOH (fraction *Sst*-1F, 11.6 mg). Fraction *Sst*-1C was applied to a reverse-phase HPLC column (TSK-gel ODS-120T; 21.5 × 300 mm, Tosoh, Tokyo, Japan) and eluted using a gradient system from CH_3CN-H_2O containing 0.2% trifluoroacetic acid (TFA) (1:9) to CH_3CN-H_2O containing 0.2% TFA (3:7) to yield the fractions *Sst*-2A (3.4 mg), *Sst*-2B (6.5 mg), *Sst*-2C (9.6 mg), *Sst*-2D (9.1 mg), *Sst*-2E (3.7 mg), *Sst*-2F (3.0 mg), *Sst*-2G (14.1 mg), *Sst*-2H (9.5 mg), *Sst*-2I (14.5 mg), *Sst*-2J (7.8 mg), *Sst*-2K (5.6 mg), *Sst*-2L (6.9 mg), *Sst*-2M (6.8 mg), *Sst*-2N (9.1 mg), *Sst*-2O (8.2 mg), *Sst*-2P (13.0 mg), *Sst*-2Q (25.7 mg), *Sst*-2R (45.1 mg), *Sst*-2S (16.4 mg), and *Sst*-2T (13.0 mg). Fraction *Sst*-1D was applied to a reverse-phase HPLC column (TSK-gel ODS-

120T; 21.5 × 300 mm, Tosoh, Tokyo, Japan) and eluted with a gradient system from $CH_3CN–H_2O$ containing 0.2% TFA (1:4) to CH_3CN-H_2O containing 0.2% TFA (3:7) to yield fractions *St*-3A–3G (total 118.0 mg), *Sst*-3H+I (98.0 mg), and *Sst*-3J–3K (29.7 mg).

Dried *S. fortunei* (61 g) was extracted with acetone-H_2O (4:1) at room temperature for 1 week. After concentration, 10.9 g of the rough extract was obtained. The extract was dissolved in H_2O, and the solution was applied to a porous polymer gel (Diaion HP-20, 200 g) open column and eluted with H_2O (fraction *Sf*-1A, 5.29 g), MeOH/H_2O (1:4) (fraction *Sf*-1B, 316 mg), MeOH/H_2O (2:3) (fraction *Sf*-1C, 362 mg), MeOH-H_2O (3:2) (fraction *Sf*-1D, 745 mg), MeOH-H_2O (9:1) (fraction *Sf*-1E, 161 mg), and MeOH (fraction *Sf*-1F, 30.1 mg). Fraction *Sf*-1C was applied to a reverse-phase HPLC column (TSK-gel ODS-120T; 21.5 × 300 mm, Tosoh, Tokyo, Japan) and eluted with a gradient system from CH_3CN-H_2O containing 0.2% TFA (1:4) to CH_3CN-H_2O containing 0.2% TFA (3:7) to yield the fractions *Sf*-2A–2G (total 138.5 mg), *Sf*-2H (22.5 mg), and *Sf*-2I (6.3 mg). Fraction *Sf*-1D was applied to a reverse-phase HPLC column (TSK-gel ODS-120T; 21.5 × 300 mm, Tosoh, Tokyo, Japan) and eluted with a gradient system from $CH_3CN–H_2O$ containing 0.2% TFA (1:4) to $CH_3CN–H_2O$ containing 0.2% TFA (3:7) to yield the fractions *Sf*-3A–3F (total 116.3 mg), *Sf*-3G (44.7 mg), and *Sf*-3H (16.0 mg).

Dried *S. nipponica* (230 g) was extracted with acetone-H_2O (4:1) at room temperature for 1 week. After the extract was concentrated, 13.2 g of the rough extract was obtained. The extract was dissolved in H_2O, and the solution was applied to a porous polymer gel (Diaion HP-20, 200 g) open column and eluted with H_2O (fraction *Sn*-1A, 5.29 g), MeOH–H_2O (1:4) (fraction *Sn*-1B, 156 mg), MeOH-H_2O (2:3) (fraction *Sn*-1C, 299 mg), MeOH-H_2O (3:2) (fraction *Sn*-1D, 438 mg), MeOH-H_2O (9:1) (fraction *Sn*-1E, 230 mg), and MeOH (fraction *Sn*-1F, 14.5 mg). Fraction *Sn*-1C was subjected to reverse

phase HPLC column (TSK-gel ODS-120T; 21.5 × 300 mm, Tosoh, Tokyo, Japan) and eluted with a gradient system from CH_3CN-H_2O containing 0.2% TFA (1:4) to CH_3CN-H_2O containing 0.2% TFA (3:7) to yield the fractions *Sn*-2A–2D (total 187.3 mg), *Sn*-2E (7.5 mg), *Sn*-2F (2.4 mg). Fraction *Sn*-1D was applied to a reverse-phase HPLC column (TSK-gel ODS-120T; 21.5 × 300 mm, Tosoh, Tokyo, Japan) and eluted with a gradient system from CH_3CN–H_2O containing 0.2% TFA (1:4) to CH_3CN–H_2O containing 0.2% TFA (3:7) to yield fractions *Sn*-3A–3E (total 217.0 mg), *Sn*-3F (23.5 mg), and *Sn*-3G (3.6 mg).

Dried *S. cortusifolia* (19 g) was extracted with acetone-H_2O (4:1) at room temperature for 1 week. After the extract was concentrated, 3.0 g of the rough extract was obtained. The extract was dissolved in H_2O, and the solution was applied to a porous polymer gel (Diaion HP-20, 50 g) open column and eluted with H_2O (fraction *Sc*-1A, 1.53 g), MeOH-H_2O (1:4) (fraction *Sc*-1B, 176 mg), MeOH-H_2O (2:3) (fraction *Sc*-1C, 344 mg), MeOH-H_2O (3:2) (fraction *Sc*-1D, 217 mg), MeOH-H_2O (9:1) (fraction *Sc*-1E, 52.8 mg), and MeOH (fraction *Sc*-1F, 27.9 mg). Fraction *Sc*-1C was applied to a reverse-phase HPLC column (TSK-gel ODS-120T; 21.5 × 300 mm, Tosoh, Tokyo, Japan) and eluted with a gradient system from CH_3CN-H_2O containing 0.2% TFA (1:4) to CH_3CN-H_2O containing 0.2% TFA (3:7) to yield fractions *Sc*-2A–2D (total 235.6 mg), *Sc*-2E (15.2 mg), and *Sc*-2F (2.9 mg). Fraction *Sc*-1D was applied to a reverse-phase HPLC column (TSK-gel ODS-120T; 21.5 × 300 mm, Tosoh, Tokyo, Japan) and eluted with a gradient system from CH_3CN-H_2O containing 0.2% TFA (1:4) to CH_3CN-H_2O containing 0.2% TFA (3:7) to yield the fractions *Sc*-3A–3E (total 112.9 mg), *Sc*-3F (24.4 mg), and *Sc*-3G (9.7 mg).

Dried *S. rebunshirensis* (19 g) was extracted with acetone-H_2O (4:1) at room

temperature for 1 week. After the extract was concentrated, 0.77 g of the rough extract was obtained. The extract was dissolved in H_2O, and the solution was applied to a porous polymer gel (Diaion HP-20, 50 g) open column and eluted with H_2O (fraction *Sr*-1A, 278 mg), MeOH–H_2O (1:4) (fraction *Sr*-1B, 39.8 mg), MeOH-H_2O (2:3) (fraction *Sr*-1C, 201 mg), MeOH-H_2O (3:2) (fraction *Sr*-1D, 45.8 mg), MeOH-H_2O (9:1) (fraction *Sr*-1E, 10.6 mg), and MeOH (fraction *Sr*-1F, 8.7 mg). Fraction *Sr*-1C was applied to a reversed-phase HPLC column (TSK-gel ODS-120T; 21.5 × 300 mm, Tosoh, Tokyo, Japan) and eluted with a gradient system from CH_3CN-H_2O containing 0.2% TFA (1:4) to CH_3CN-H_2O containing 0.2% TFA (3:7) to yield fractions *Sr*-2A–2D (total 140.4 mg), *Sr*-2E (7.5 mg), and *Sr*-2F (2.4 mg).
